# Supplementary material for: Spatio-temporal modelling of COVID-19 infection and associated risk factors in Dakar, Senegal
Source: PLOS Glob Public Health. 2026 Jun 11;6(6):e0004945. doi: 10.1371/journal.pgph.0004945 (PMC13257971; doi:10.1371/journal.pgph.0004945)
Supplement: Attachment — Submitted filename: PGPH-D-25-01352_R1_reviewer_AD_comments.pdf [file pgph.0004945.s008.pdf]

PLOS Global Public Health

Spatio-temporal modelling of COVID-19 infection and associated risk factors in Dakar, Senegal

--Manuscript Draft--

**Manuscript Number:**	PGPH-D-25-01352R1
**Article Type:**	Research Article
**Full Title:**	Spatio-temporal modelling of COVID-19 infection and associated risk factors in Dakar, Senegal
**Short Title:**	COVID-19 infection and associated risk factors in Dakar, Senegal.
**Corresponding Author:**	Assane Niang Gadiaga, Ph.D<br>University of Southampton - Highfield Campus: University of Southampton<br>Southampton, Hampshire UNITED KINGDOM OF GREAT BRITAIN AND NORTHERN IRELAND
**Order of Authors:**	Assane Niang Gadiaga, Ph.D
	Mame Wodji Tine
	Aminata Niang Diene, Professor
	Catherine Linard, Professor
	Niko Speybroeck, Professor
	Ortis Yankey, Researcher
	Somnath Chaudhuri, Researcher
	Chibuzor Christopher Nnanatu, Senior Researcher
	Eimear Cleary, Senior Researcher
	Shengjie Lai, Principal Research Fellow
	Attila Nando Lazar
	Andrew James Tatem, Professor
**Keywords:**	COVID-19 infection; Bayesian modelling; Dakar; contextual risk factors
**Abstract:**	The spread of infectious diseases is a major threat to global health and economy and the recent COVID-19 pandemic is a perfect illustration of this. Appropriately modelling and accurate prediction of the outcome of disease spread over time and across space is a critical step towards informed development of effective strategies for public health interventions. In low and middle-income countries, however, the scarcity of spatially disaggregated time-series infectious diseases data often limits the analysis of the burden of infectious disease at a broad-scale, and the effects of the contextual risk factors is not often fully captured. In this study, we investigate the spatiotemporal patterns of COVID-19 infection in Dakar at the neighbourhood level, and evaluate the impact of potential risk factors. Geostatistical models based on COVID-19 infection were used to explain and predict the spatiotemporal distribution of COVID-19 infection between June 2020 and June 2021. We specified a Bayesian regression model that incorporates a spatio-temporally autocorrelated random effect in order to quantify the evolution of the spatial patterns of the COVID-19 infection overtime. Results show significant strong spatial heterogeneity but relatively small temporal variations of the COVID 19 distribution, and a positive association between adjusted population density (mean of the posterior probability: 0.29, credible interval: 0.24-0.34) and residential areas (mean of the posterior probability: 1.25, credible interval: 0.66-1.83) with COVID-19 infection. Western areas are at higher risk of COVID-19 infection compared to eastern and less densely populated peripheral neighbourhoods. Measuring the role of contextual risk factors and mapping the at-risk areas can provide valuable insights for policymakers in low- and middle-income countries, enabling more targeted public health interventions. These efforts also support the management of endemic diseases and preparedness for future outbreaks.

Powered by Editorial Manager® and ProduXion Manager® from Aries Systems Corporation

Additional Information:	
**Question**	**Response**
**Financial Disclosure**<br><br>Enter a financial disclosure statement that describes the sources of funding for the work included in this submission and the role the funder(s) played. This includes grants and any commercial funding of the work or authors.<br><br>This statement will be typeset if the manuscript is accepted for publication.<br><br>*Please review the submission guidelines and the instructions link below for detailed requirements and guidance.*	This work was supported in part by the Gates Foundation under the GRID3 project (INV-044979). The conclusions and opinions expressed in this work are those of the author(s) alone and shall not be attributed to the Foundation. Under the grant conditions of the Foundation, a Creative Commons Attribution 4.0 License has already been assigned to the Author Accepted Manuscript version that might arise from this submission. Please note works submitted as a preprint have not undergone a peer review process. Andy Tatem and Shengjie Lai were supported by the National Institutes of Health (R01AI160780) under the MIDAS project.
**Competing Interests**<br><br>On behalf of all authors, disclose any competing interests that could be perceived to bias this work.<br><br>This statement will be typeset if the manuscript is accepted for publication.<br><br>*Please review the instructions link below and PLOS Global Public Health's competing interests policy to determine what information must be disclosed at submission.*	The authors declare no competing interests.
**Data Availability**<br><br>Before publication, Authors are required to make fully available and without restriction all data underlying their findings. Please see our PLOS Data Policy page for detailed information on this policy.<br><br>A **Data Availability Statement**, detailing	The epidemiological data used in this study is publicly available through a request from the Ministry of Health. The geospatial predictors that we utilised to perform the analyses are freely available through the WorldpPop (https://www.worldpop.org/datacatalog/) and MAUPP data repositories (https://maupp.ulb.ac.be/page/grippa2018/).

where the data can be accessed, is required at first submission. Insert your Data Availability Statement in the box below.<br><br>Please see the data reporting section of our submission guidelines for instructions on what you need to include in your Data Availability Statement.<br><br>This statement will be typeset if the manuscript is accepted for publication.<br><br>*PLOS allows rare exemptions to address legal and ethical concerns. If you have legal or ethical restrictions, please use the box below to detail these in full sentences for the Journal team to consider.*	

Manuscript
Click here to access/download;Manuscript;Manuscript.docx

Spatio-temporal modelling of COVID-19 infection and associated risk factors in Dakar, Senegal

Authors and affiliation:

Assane N. Gadiaga[1,2*],, Mame Wodji Tine[2,3], Aminata Niang Diene[2,3], Catherine Linard[4], Niko Speybroeck[5], Ortis Yankey[1], Somnath Chaudhuri[1], Chibuzor Christopher Nnanatu[1], Eimear Cleary[1], Shengjie Lai[1], Attila N. Lazar[1], Andrew J. Tatem[1]

[1]WorldPop, Department of Geography and Environment, University of Southampton, Southampton, UK.

[2]Laboratoire dynamiques territoriales et sante, Université Cheikh Anta Diop de Dakar, Dakar, Senegal.

[3]Department of Geography, Université Cheikh Anta Diop de Dakar, Dakar, Senegal.

[4]Department of Geography, Université de Namur, Namur, Belgium.

[5]Université catholique de Louvain, Louvain-la-Neuve, Belgium.

[*]Corresponding author

Abstract

The spread of infectious diseases is a ma[illegible]or threat to global health and economy and the recent COVID-19 pandemic is a perfect illustration of this. Appropriately modelling and accurate prediction of the outcome of disease spread over time and across space is a critical step towards informed development of effective strategies for public health interventions. In low and middle-income countries, however, the scarcity of spatially disaggregated time-series infectious diseases data often limits the analysis of the burden of infectious disease at a broad-scale, and the effects of the contextual risk factors is not often fully captured. In this study, we investigate the spatiotemporal patterns of COVID-19 infection in Dakar at the neighbourhood level, and evaluate the impact of potential risk factors. Geostatistical models based on COVID-19 infection were used to explain and predict the spatiotemporal distribution of COVID-19 infection between June 2020 and June 2021. We specified a Bayesian regression model that incorporates a spatio-temporally autocorrelated random effect in order to quantify the evolution of the spatial patterns of the COVID-19 infection overtime. Results show significant strong spatial heterogeneity but relatively small temporal variations of the COVID 19 distribution, and a positive association between adjusted population density (mean of the posterior probability: 0.29, credible interval: 0.24-0.34) and residential areas (mean of the posterior probability: 1.25, credible interval: 0.66-1.83) with COVID-19 infection. Western areas are at higher risk of COVID-19 infection compared to eastern and less densely populated peripheral neighbourhoods. Measuring the role of contextual risk factors and mapping the at-risk areas can provide valuable insights for policymakers in low- and middle-income countries, enabling more targeted public health interventions. These efforts also support the management of endemic diseases and preparedness for future outbreaks.

1. Introduction

The COVID-19 pandemic has had profound health, economic, and social consequences worldwide throughout the year 2020 until now. Since its emergence in late 2019, the disease has spread rapidly, leading to unprecedented public health responses such as lockdowns, social distancing measures, and mass vaccination campaigns [1]. As of April 2024, the COVID-19 related death toll had risen to more than 7 million from more than 700 million reported infections [2]. While widespread vaccination efforts have significantly reduced transmission and mortality [3], the containment of the virus is challenging because of the biological features of the virus [4], involving mutating variants that mitigate the effectiveness of the vaccine, leading to sustained rates of infection throughout the community.

Given the persistent risk of COVID-19 variants worldwide, assessing the disease spread and understanding its spatiotemporal patterns are key to effective monitoring, prevention and control. This is particularly true in many LMICs, where fragile disease surveillance system were unable to monitor the progress of COVID-19 and formulate appropriate responses. Recent studies have explored the role of various contextual factors in shaping the spread of COVID-19, including population density, landscape connectivity, human mobility, socioeconomic characteristics, environmental conditions, pollution and smoking [5–11]. Population density, in particular, has been widely investigated as a risk factor of disease transmission, given the fact that people's proximity to each other increases likelihood of infection [12]. In many cases, the relationship was positive, with more densely populated areas having higher rates of infections or experiencing early outbreaks [5,10,13,14]. Highly connected areas also face earlier and higher COVID 19 infection rates [15–18], and socioeconomic factors such as residential characteristics drive disparities in COVID 19 cases and deaths [7,19–21].

Several statistical approaches have been used to investigate the role of the aforementioned risk factors on the spread of the COVID-19 disease. They are either classical and frequentist methods, which remain at the core of most practical statistical applications [22], or Bayesian methods, which have been increasingly used in the field of spatial epidemiology due to their ability to incorporate spatial and temporal dependencies [23,24].

Although the scientific community is deploying efforts to understand contextual factors invloved in the spread of COVID-19, existing epidemiological data are not often resolved at spatial levels that allows for in-depth analyses; they are in most cases available at a large scale (i.e., regional or national levels) and the patterns of the disease at finer levels (i.e., at the intra urban levels) remains unknown.

In Senegal, the first case of COVID-19 was reported in early March 2020, and the majority of subsequent Senegalese cases were reported in Dakar, the capital city. According to the Ministry of Health, as of May 29, 2020, the Dakar region accounted for nearly three quarter (2525 cases out of the 3429 detected in the country; 73.6%) of reported COVID-19 infections in Senegal [25]. Public health authorities classified the source of the COVID-19 infection as one of the following four categories: (1) contact cases; (2) community cases; (3) imported cases and (4) evacuated cases. An infection was termed as a community case when the source of infection was unknown [26], making efforts to control the pandemic more challenging. Against this backdrop, the present study analysed the spatiotemporal dynamics of COVID-19 disease in Dakar, with a focus on the COVID-19 infection which arose from community transmission, and using COVID-19 time-series data at the finest administrative level.

Through a spatio-temporal Bayesian framework we evaluate the impact of population density, temperature, distance to marketplaces, non-pharmaceutical interventions such as ease of social distancing index, and residential characteristics on COVID-19 spread. Distance to marketplaces is used here as a proxy of mobility [27], while residential and non-residential areas indicates differences in living conditions and is a useful proxy on poverty levels [28]. In addition to the widely used population density (count of people per total area), adjusted population density that refers to the count of people per built-up area was also considered. This approach seeks to better capture the effect of crowding, because, although density expresses a ratio between population and an unit of area, these units are not uniformly distributed over the space. Our aim is to understand the spatiotemporal dynamic of COVID-19 spread in Dakar as well as related potential drivers. In a context of fragile health system, as in many low- and middle-income countries, having detailed time-series epidemiological data at the finest administrative level for Dakar offers a unique opportunity to explore advanced spatial statistical modelling approach and improve our understanding of localised transmission patterns. Understanding these localised transmission patterns is essential for designing targeted public health interventions, optimizing resource allocation, and mitigating the impact of future outbreaks.

2. Materials and methods

2.1. Study area and data

2.1.1. Study area

Dakar is the capital city of Senegal (Fig 1) and an example of African city that combines several aspects of the Africa's urban growth: rapid urbanization, land cover and land use changes, inequalities in living conditions [29] that have led to different health status [28]. Located in the westernmost part of the African continent (Fig 1), Dakar is the driving force of the Senegalese economy and through its markets, banks and industrial sector remains a magnet for migrants from all over the country. According to the last national census conducted in 2023, the region had a population of 4 million inhabitants, nearly a quarter of the Senegalese population (22 %) in an area representing only 0.3 % of the country [30]. The availability of georeferenced fine scale census information [28] and high quality, very-high resolution remote sensing land cover and land use products [31,32] has improved our knowledge on health inequalities and associated socioeconomic and environmental risk factors [28,33].

Fig 1. Geographical location of Dakar.

2.1.2. Data

The epidemiological data used in this study were obtained from the daily reports of COVID-19 infection in Dakar produced by the Ministry of health [25]. Data cover the period from 01/06/2020 to 30/06/2021 and records the number of confirmed COVID-19 infections from community-based transmission. Each anonymised record includes the date of the confirmed infection, the location of the infection and the number of new infections. There can be more than one record per date, with similar or different locations. The location of the infection often refers to the neighbourhood, which is the smallest administrative unit in an urban area. However, in some situations, the location can refer to a higher administrative extent, i.e. the commune, or a lower level spatial extent such as the housing unit. Geographic coordinates of each infection record were obtained by joining the excel-based disease data

file with the neighbourhood shapefile. To do this, the unique identifier availabe in the latter was assigned to the disease record when there is a matching between the location of the infection and the polygon shapefile. When the location of the infection was referring to a Commune or a housing unit, a fuzzy-matching algorithm was used to merge the infection record with the neighbourhood shapefile. After the geocoding of the infection records, the daily cases were aggregated by month for each neighbourhood. Thus, each neighbourhood has an aggregated count spanning the study period, providing an idea of the basic severity status of the neighborhood during the pandemic (covid19_Dakar). Although the neighborhood-level data offer an opportunity to examine the intra-urban variation of COVID-19 infection in Dakar, it is important to note that this does not provide a complete picture of the status of the pandemic in the city, as only infections from community transmission are analysed. In fact, only community based transmission can be used to reconstruct the spatio-temporal dynamic of the pandemic because, unlike the other type of infection, it includes information about the location of the infection (patient's residence).

Furthermore, just as there are multiple records for some dates, there are also days with no reported infections from community transmission. This might weaken the quality of the study, although it remains unclear whether the absence of reported infections is due to a lack of such cases among tested patients on the corresponding dates. The cumulative incidence and temporal distribution of COVID-19 infections over the study period are illustrated in Figs 2 and 3, respectively.

Fig 2. Aggregated incidence of COVID-19 confirmed infection from community transmission from June 2020 to June 2021.

Fig 3. Monthly distribution of COVID-19 infection from community transmission in Dakar from June 2020 to June 2021.

As predictors of COVID-19 infection, we used two high resolution satellite derived land use [32] and gridded population datasets [34], with a spatial resolution of 10m for the population data and street block level for the land use map. Additionally, distance to marketplaces, ease of social distancing index, and temperature data obtained from the WorldPop data repository were used [35]. The land use map depicts several spatial metrics, such as residential built-up areas (planned or unplanned), non-residential built-up areas (administrative, commercial and service areas), agricultural vegetation, natural vegetation, water bodies and baresoil areas. It is a precise representation of the Dakar's urban landscape, with an overall accuracy of 79 % [32] and regarding its usefulness in understanding inequalities in health and living conditions [28,33]. In the present study, however, this accuracy may be hampered by the mismatch between the year the satellite data were collected (2015) and the year in which the COVID-19 data were obtained (2020-2021). Although built and settlement layers are time-variant, we assume that this 5 year gap is negligible in the particular context of Dakar. To further assess this, we used the world settlement footprint data [36] and calculated the average percentage of change in the built-up areas at the neighbourhood level from 2015 to 2019. On average, built-up areas per neighbourhood have grown by 5% within the period 2015-2019 in Dakar, indicating minimal changes. It is important to note that the land use data does not cover Goree and Ngor islands as well as some parts of eastern Dakar. Thus, to ensure a full overlap between the COVID-19 data and the

geospatial predictors, the spatial extent of the study area was reduced and sparsely built-up areas in the eastern urbanisation front were excluded from the analysis.

Using these remotely sensed data sources, we first extracted the average residential built-up areas, non-residential built-up areas, temperature, and distance to marketplaces for each neighborhood. The total gridded population per neighborhood was divided by the neighborhood's total area to calculate population density. We then computed the adjusted population density by dividing the gridded population counts by the total built-up area for each neighborhood. Additionally, built-up area was divided by the neighborhood's total area to calculate built-up density. The covariates used for the modeling are listed in Table 1.

Table 1. List of covariates used for the modelling

Variable	Description	Source
**Temperature**	Annual average temperature	WorldPop
**Built-up density**	Built-up area per total area	MAUPP[a]
**Distance to marketplaces**	Average distance to marketplaces	WorldPop
**Population density**	Count of people per total area	MAUPP
**Adjusted population density**	Count of people per built-up area	MAUPP
**Residential areas**	Proportion of land use (LU) used for residential purposes	MAUPP
**Non-residential ACS areas**	Proportion of LU without residences used for administrative, commercial and services (ACS) purposes	MAUPP
**Ease of social distancing index**	Score index that evaluates the feasibility of social distancing measure	WorldPop

[a]MAUPP = Modelling and forecasting African Urban Population Patterns for vulnerability and heath assessments (https://maupp.ulb.ac.be/).

Statistical analyses were performed to check for multicollinearity among the covariates and to remove any covariates that were not statistically significant. To address this, we used the Variance Inflation Factor (VIF) to measure the level of collinearity. Covariates with a VIF greater than 5 were considered to have high multicollinearity and were removed from the analysis [37,38]. Only covariates with a VIF less than 5 and those that were statistically significant were selected for the final model. Among the 8 variables considered for this analysis and introduced in Table 1, built-up density and ease of social distancing index were found showing multicollinearity issue and further dropped. In the following section, the Bayesian statistical modelling approach we developed to conduct this research is presented.

2.1.3. Ethics statements

The epidemiological datasets used in this study were completely anonymised and aggregated to administrative level to avoid confidentiality issues in accordance with relevant data protection regulations. The study adhered to the Declaration of Helsinki as it ensures respect for all participants

and protect their privacy, health and rights. Additionally, the data has been made publicly available in the official website of the Ministry of Health through a web-based visulisation platform.

2.2. Modelling COVID-19 infection risk

The spread of infectious diseases like COVID-19, involves complex patterns over both space and time. This complexity arises from the interactions between human behaviour, environmental factors, and disease transmission. To analyse COVID-19 cases in Dakar, Senegal, we have used a Bayesian spatiotemporal modelling approach. This method allows us to account for both spatial and temporal autocorrelation structures in the data. Spatial autocorrelation refers to the way disease cases are clustered in certain areas, while temporal autocorrelation deals with how cases change over time. Our modelling approach is important because COVID-19 cases are not evenly distributed across different locations or time periods, as illustrated in Figs 2 and 3.

In this study, we applied three distinct modelling approaches to analyse the spread of COVID-19 in Dakar. Our first approach focused on identifying monthly trends across the entire city. For this, we used both first-order and second-order random walk models (RW1 and RW2) to capture temporal randomness. To account for neighbourhood-level variations, we included an independent and identically distributed (IID) random effect. In this approach, we aggregated the total number of COVID-19 cases across Dakar for each month, using these monthly totals as the response variable.

In the second approach, we examined COVID-19 case counts at the neighbourhood level, capturing spatial variations within Dakar in addition to temporal trends. This separable spatiotemporal model included spatial randomness based on individual neighbourhoods, temporal randomness, and the IID effect of each neighbourhood. Finally, our third approach also involved spatiotemporal modelling, but with an added spatiotemporal interaction effect. This term represented an unobserved independent effect for each combination of neighbourhood and month, allowing us to capture unique patterns in each area over time. The rationale for constructing a temporal regression and spatiotemporal approaches is that temporal patterns often occurs in different aspects of health, particularly in infectious disease transmission [39]. Furthermore, infectious disease diffusion and spread is an intrinsically spatial process, as it involves complex interactions between human and different landscapes attributes. Therefore, the motivation for constructing multiple models in this study, and comparing them is that, in addition to temporal correlation, possible spatiotemporal correlation may play a crucial role, with the spatial correlation constant or changing over time.

2.2.1. Temporal modelling of COVID-19 infection

In the first approach, we have employed a temporal Poisson regression framework to analyse the temporal dynamics of COVID-19 in Dakar. In this model, the number of confirmed cases at each time point was represented using a Poisson likelihood in that we are dealing with a count data. The temporal resolution of the analysis has been set to one month, allowing us to explore trends and changes in the number of infected cases over time. Additionally, we incorporated geospatial variables to assess their potential correlations with the infection rates.

Let Y_t and E_t represent the number of observed and expected COVID-19 cases in the population at time t, where $t = 1, \dots, T$. We assume that, given the relative risk q_t the number of observed cases follows a Poisson distribution:

$$Y_t \mid q_t \sim \text{Poisson} \; (\lambda_t = E_t \, q_t)$$

where E_t is the total population for the study area, and the log-risk is modelled as:

$$\log(q_t) = \beta_0 + Z_t^T \beta + u_i + \gamma_t \; \dots\dots\dots \; (1)$$

where, Z_t represents the matrix of covariates used to account for various factors influencing the infection rates. The term u_i is an IID random effect that captures unobserved variability across the neighbourhoods. Additionally, γ_t represents the temporal effects, which can be modelled using either a RW1 or a RW2 to account for the temporal structure and potential trends over time. In the case of the RW1, the temporal effects γ_t are modeled such that the increments $\Delta\gamma_t = \gamma_t - \gamma_{t-1}$ follow a Gaussian distribution with zero mean and precision τ. On the other hand, for a RW2, the temporal effects are modeled as $\Delta^2 \gamma_t = \gamma_t - 2\,\gamma_{t-1} + \gamma_{t-2}$ also follow a Gaussian distribution with zero mean and precision τ. This RW2 model provides a smoother temporal evolution over time compared to the RW1 model, making it particularly useful for capturing complex temporal patterns such as trends, cycles, or deviations from simple linear temporal patterns. For prior selection, we have used penalized complexity (PC) priors [39]. For the IID random effect, the PC prior on the precision was set to [0.5, 0.01], where 0.5 is the central tendency and 0.01 controls the spread or uncertainty around it. For the RW1 or RW2 model on the monthly data, the PC prior on the precision with parameters [1, 0.01] represents a location parameter of 1 and a scale parameter of 0.01, indicating a prior belief that the precision is centered around 1 with relatively low uncertainty. The prior for the coefficient vector $\beta = (\beta_0, \dots, \beta_p)$ which follows a zero-mean Gaussian distribution with a precision of 0.001. All log-precision parameters followed inverse gamma distributions with shape parameter $(\alpha = 1)$ and scale parameter $(\beta = 0.00005)$.

2.2.2. Spatiotemporal modelling of COVID-19 infection

In the second approach, we employed a spatiotemporal Poisson regression framework to analyze the spread of COVID-19 in Dakar. In this model, the number of confirmed cases at each spatial unit (neighborhood) and time point was represented using a Poisson likelihood. Similar to the first approach, the temporal resolution of the spatiotemporal analysis was set to one month, allowing us to observe trends and changes in case numbers over time (months). The spatiotemporal model included spatial random effects to account for the influence of neighboring regions on case numbers. It also incorporated temporal random effects to capture trends and seasonal variations over time. Additionally, geospatial covariates were included to explore their potential associations with infection rates.

Let Y_{it} and E_{it} represent the number of observed and expected COVID-19 infected cases in the i-th area (neighbourhood) and the t-th period (month), where $t = 1, \dots, T$. We assume that, given the relative risk q_{it} , the number of observed cases follows a Poisson distribution:

$$Y_{it} \mid q_{it} \sim \text{Poisson} \; (\lambda_{it} = E_{it} \, q_{it})$$

where, E_{it} are the total population for individual neighbourhood of the study area and the log-risk is modelled as in Equation (2) for spatiotemporal separable model and in Equation (3) for spatiotemporal interaction model.

$$\log(q_{it}) = \beta_0 + Z_{it}^T \beta_{it} + S(x_i) + u_i + \gamma_t \;\ldots\ldots\ldots\ldots\ldots\; (2)$$

$$\log(q_{it}) = \beta_0 + Z_{it}^T \beta_{it} + S(x_i) + u_i + \gamma_t + \delta_{it} \;\ldots\ldots\; (3)$$

where in both Equation (2) and (3), $S(.)$ represents the spatially structured random effects, and Z_{it} represents the matrix of covariates used in the model. The temporally structured effect has been represented by γ_t. In Equation (3), δ_{it} represents the spatio-temporal interaction between the two structured effects. The IID random effect u_i is added to the model to capture neighbourhood-level variability with a penalized complexity (PC) prior on the precision set as $[0.5{,}0.01]$ where we assumed a prior mean of 0.5 and a prior precision of 0.01. The matrix of covariates was built by stacking the covariate values for every space-time combination, and separate random effect mapping matrices link these observations to spatial, temporal or spatiotemporal latent effects. We have modelled temporal trends using a RW1 for the monthly data. This model was scaled for identifiability, and we applied a PC prior on the precision with parameters [1,0.01] with a prior mean of 1 and a prior precision of 0.01. For all prior selections, we used PC priors following the framework proposed by Simpson et al. [39]. We have assigned a vague prior to the vector of coefficients $\beta = \left(\beta_0, \ldots, \beta_p\right)$ which follows a zero-mean Gaussian distribution with a precision of 0.001. Finally, all parameters associated with log-precisions are assigned inverse Gamma distributions with shape parameter $(\alpha = 1)$ and scale parameter $(\beta = 0.00005)$. The default prior distributions for all parameters in R-INLA were selected based on commonly used priors in previous studies [40–42]. Our findings demonstrate robustness to alternative priors. We have tested multiple scenarios with different priors and consistently obtained the same results. As shown in Equation (3), we introduced a spatiotemporal interaction effect as an unobserved, independent term for each region-period combination (i, t), modelled as $\delta_{it} \sim Normal\left(0, \frac{1}{\tau\delta}\right)$. This interaction term assumes no additional structure, representing independent variations from the main spatial and temporal effects when those are included in the model. It captures global space-time heterogeneity and is typically modeled as white noise, as discussed in previous studies [40]. This interaction term offers a more flexible structure, capturing localized deviations from the main spatial and temporal patterns. To compare modelling strategies, we constructed the third model set that incorporate all covariates, using both spatiotemporal interactions and PC priors. A summary of these competing models is presented in Table 2 in the Results Section.

The dataset used in this study shows zero inflation. As such, zero-inflated Poisson and Poisson Hurdle models can be used to accommodate zero-inflated discrete distributions. While ZIP models add extra probability mass to the zero outcome [43], Poisson hurdle models, on the other hand, are characterized by a mixture of zero and non-zero outcomes [44,45]. In a ZIP model, the response variable Y_{it} is zero with probability π, and follows a Poisson distribution with parameter λ_{it} with probability $1 - \pi$. In particular,

$$Y_{it} = \begin{cases} 0, & \text{with prob } \pi + (1-\pi)e^{-\lambda_{it}} \\ k, & \text{with prob } (1-\pi)\,\frac{\lambda_{it}^{k} e^{-\lambda_{it}}}{k!},\ k \geq 1 \end{cases} \quad \text{......... (4)}$$

Conversely, in a Poisson hurdle model, the response variable Y_{it} is zero with probability π, and follows a truncated Poisson distribution with parameter λ_{it} with probability $1-\pi$. Mathematically,

$$Y_{it} = \begin{cases} 0, & \text{with prob } \pi \\ k, & \text{with prob } \frac{(1-\pi)}{1-e^{-\lambda_{it}}}\left(\frac{\lambda_{it}^{k} e^{-\lambda_{it}}}{k!}\right),\ k \geq 1 \end{cases} \quad \text{......... (5)}$$

In the model fitting process, Bayesian inference typically employs Markov Chain Monte Carlo (MCMC) methods to estimate the joint posterior distribution of model parameters [46,47]. But for big dataset such as the time-series data used in this study, this can be computationally intensive. As a faster alternative, the integrated nested Laplace approximation (INLA) approximates posterior marginal distributions efficiently, especially for models represented as latent Gaussian Markov random fields (GMRF) [42,48]. In the current study, we have applied INLA along with a stochastic partial differential equation (SPDE) method. The SPDE method allows us to model a continuous spatial process, such as a Gaussian field (GF), using a discretely indexed spatial random process like a GMRF [49,50].

In particular, the spatial random process represented by $S(.)$ explicitly denote dependence on the spatial field, follows a zero-mean Gaussian process with Matérn covariance function [51] represented as:

$$\mathrm{Cov}\left(\mathrm{S}(\mathrm{x_i}), \mathrm{S}(\mathrm{x_j})\right) = \frac{\sigma^2}{2^{\nu-1}\Gamma(\nu)}\left(\kappa \parallel \mathrm{x_i} - \mathrm{x_j} \parallel\right)^{\nu} \mathrm{K}_{\nu}\left(\kappa \parallel \mathrm{x_i} - \mathrm{x_j} \parallel\right)$$

The parameter σ^2 represents the process variance, indicating the variability of the values around the mean. The smoothness parameter $\nu > 0$ influences the rate of decay of correlations with distance, while the scaling parameter $\kappa > 0$ governs the overall range of the covariance. The term $\parallel \mathrm{x_i} - \mathrm{x_j} \parallel$ denotes the Euclidean distance between locations. The modified Bessel function of the second kind, $K_{\nu}(.)$, defines the covariance structure, and $\Gamma(\nu)$ is the gamma function, normalizing the covariance for scaling purposes.

The parameterized model we follow is of the form:

$$(\kappa^2 - \Delta)^{(\alpha/2)}(\tau S) = W \text{ on } \mathbb{R}^d$$

where $\Delta = \sum_{i=1}^{d} \frac{\partial^2}{\partial x_i^2}$ is the Laplacian, $\alpha = (\nu + d/2)$ is the smoothness parameter, τ is inversely proportional to σ, W is Gaussian white noise and $\kappa > 0$ is the scale parameter, related to range r, defined as the distance at which the spatial correlation becomes negligible. For each ν, we have $r = \sqrt{8\nu}/\kappa$, with r corresponding to the distance where the spatial correlation is close to 0.1. Note that we have $d = 2$ for a two-dimensional process, and we fix $\nu = 1$, so that $\alpha = 2$ in this case [40–42]. In the current study, we have applied the PC priors [39] for the Matérn model, which helps prevent overfitting by supporting simpler models. The prior for the marginal standard deviation is set as $[1,0.1]$ where we assumed a prior mean of 1 and a prior precision of 0.1. This allows some flexibility around a mean of 1 based on our expectations of spatial variation. We utilized the centroids of each neighborhood as the target locations for SPDE mesh construction. According to Verdoy [52], an optimal mesh should possess a sufficient number of vertices to facilitate effective predictions, and within a limit to have control over computational time. Following this principle, the triangulated mesh has been generated using inla.mesh.2d function from R-INLA package, based on Delaunay triangulation method [50], with the best fitting mesh having 7365 vertices for the 1078 neighbourhoods considered in this

study. S1 Fig depicts the SPDE mesh designed for the study area with 1078 neighborhood centroid locations highlighted as red points and the blue line marks the boundary of the study area. We have created a projection matrix to transfer the spatially continuous Gaussian random field from the observations to the mesh nodes. The centroids of the neighbourhoods and the triangulation in the mesh are used to develop this projection matrix.

All models assume that the number of COVID-19 cases follows a Poisson likelihood, with the expected count determined by population size and relative risk. The models differ in how spatial and temporal dependencies are incorporated. The temporal model includes monthly random effects modelled as either RW1 or RW2 processes, while the spatiotemporal models incorporate both spatially structured effects (using SPDE approach) and temporally structured effects (RW1). The interaction model further includes an independent spatiotemporal interaction term to capture unobserved localized deviations. Estimated parameters are inferred from the data through posterior distributions and include the regression coefficients (β), IID random effects (u_i), temporal effects (γ_t), spatiotemporal interactions (δ_{it}), and spatial field values ($S(x_i)$). Assumed parameters are chosen based on prior knowledge or literature, such as the parameters of the PC priors ([0.5, 0.01] for u_i, [1, 0.01] for γ_t) or the prior for the SPDE spatial range [40]. Fixed parameters are held constant and define model structure, such as the Matérn smoothness parameter ν and the spatial dimension [50]. A summary of estimated parameters and priors is presented in table 2.

Table 2. Estimated parameters for the spatiotemporal models.

Parameter	Description	Model(s) Used	Prior Distribution
β_0, β	Fixed effects (intercept, covariates)	All models	Normal(0, 0.001)
u_i	IID random effect (neighbourhood-level variability)	Spatiotemporal	PC prior [0.5, 0.01]
γ_t	Temporal random effect (RW1 or RW2)	Temporal, Spatiotemporal	PC prior [1, 0.01]
δ_{it}	Spatiotemporal interaction term	Interaction model only	Normal(0, $1/\tau\delta$)
$S(x_i)$	Spatially structured effect (SPDE)	Spatiotemporal	Matérn covariance, PC prior [1, 0.1]
τ	Precision parameters (for random effects)	All models	Inverse Gamma(1, 0.00005)
ν	Smoothness parameter in SPDE	Spatiotemporal	Fixed at 1
κ	Scale parameter in SPDE	Spatiotemporal	Derived from range r

In the model fitting process, we have included eight covariates, such as population density, adjusted population density, built-up density, average value of temperature, residential built-up areas, non-residential built-up areas, distance to marketplaces, ease of social distancing index, as outlined in Section 2.1. The variable month spans from 1 to 12, representing each month of the year, and month is treated as factor in the model. We used data on COVID-19 cases from June 2020 to May 2021 (a total of 12 months) to fit the model described in Equation (2). To assess prediction accuracy, we held out data from the following month, June 2021, as a test set. Using this fitted model, INLA then predicted the COVID-19 cases for these held-out months by computing the predictive distributions based on the posterior distributions of the model parameters and latent variables. This approach allows INLA to provide both point predictions and uncertainty estimates, making it suitable for evaluating model accuracy on out of sample data. Finally, we compared the predicted values with the original observed COVID-19 cases from June 2021 to evaluate the predictive performance of the model, with detailed comparison results and performance metrics discussed in Section 3. All the analyses were conducted in R statistical software (v.4.2.2).

3. Results

The following results summarize the outcomes of our Bayesian spatiotemporal modelling approaches, highlighting the temporal dynamics, spatial heterogeneity, and zero-inflation characteristics of COVID-19 case distributions in Dakar. For the latter, a ZIP hurdle distribution is used in the modelling, and the parameter significance as well as the goodness of fit of the three fitted Bayesian ZIP hurdle models are presented in Table 3. It appears that the spatiotemporal interaction model was the best model to fit the data. Indeed, the best fitting spatiotemporal interaction model (M3) has the lowest Deviance Information Criterion (DIC) and Watanabe-Akaike information Criterion (WAIC) values compared to the temporal (M1) and separable spatiotemporal model (M2). The DIC and WAIC values varied slightly from the temporal to the separable spatial-temporal models (37050 and 37049 DIC values for M1 and M2, respectively, then 37061 and 37060 WAIC values for M1 and M2, respectively). When a spatiotemporal interaction is specified, the DIC and WAIC has significantly dropped, indicating higher predictability when the space-time interaction is accounted for.

Table 3. Posterior estimates of model parameters of temporal and spatiotemporal models of COVID-19 in Dakar.

			Spatiotemporal Model			
	Temporal Model (M1)		Separable Model (M2)		Interaction model (M3)	
**Variable**	**Mean**	**95% BCI [a]**	**Mean**	**95% BCI**	**Mean**	**95% BCI**
**Intercept**	-10.23	-55.38,-34.91	-10.25	-55.42,34.92	-10.27	-55.46,34.92
**Residential areas**	1.25	0.67,1.83	1.25	0.67,1.83	1.25	0.66,1.83

**Non-residential areas**	-0.66	-1.29,-0.04	-0.66	-1.29,-0.04	-0.67	-1.29,-0.04
**Population density**	-0.003	-0.003,-0.002	-0.003	-0.003,-0.002	-0.003	-0.003,-0.002
**Adjusted population density**	0.29	0.24,0.34	0.29	0.24,0.34	0.29	0.24,0.34
**Temperature**	0.03	-0.12,-0.18	0.03	-0.12,0.18	0.03	-0.12,0.18
**Distance to marketplaces**	0.00	0.00,0.00	0.00	0.00,0.00	0.00	0.00,0.00
**DIC [b]**	37044		37043		36711	
**WAIC [c]**	37056		37055		36885	

[a] BCI: Bayesian Credible Interval

[b] DIC: Deviance Information Criterion

[c] WAIC: Watanabe-Akaike Information Criterion

For the models specified in Equations (2) and (3), we examine the posterior distributions of fixed and random effects for both the separable spatiotemporal model and the spatiotemporal interaction model. These posterior distributions are presented in S2 and S3 Figs, and S4 and S5 Figs. For both models, the posterior distribution of the fixed effects provides insights into the impact of covariates on the outcome across space and time.

The results indicate that the covariates month number, distance to marketplace, and temperature are non-significant in both models, with null posterior mean or zero included within the range of the 95% credible interval. Conversely, population density and non-residential ACS areas show a significant negative effect, suggesting a negative association in the model. As the proportion of non-residential areas, and the count of people per hectare per neighbourhood's total area increase, the expected mean of the COVID-19 infection decrease by 0.003 and 0.67, respectively. These expected values fall within the 95% credible interval for both the effect of non-residential areas (-1.29, -0.04) and population density (-0.003, -0.002). Adjusted population density and residential areas exhibit a significant positive effect, indicating a strong positive influence. When the proportion of residential areas, and the count of people per built-up area increase, the expected mean of COVID-19 infection increase by 0.29 and 1.25, respectively. Also there is a 95% probability this effect lies between 0.66 and 1.83 for residential areas, and between 0.24 and 0.34 for adjusted population density. For both spatiotemporal models, the fixed effects demonstrate a similar trend and nature.

On the other hand, the posterior distribution of the random effects captures underlying spatial, temporal, and spatiotemporal variability. In the separable model, spatial and temporal effects are modeled independently, assuming no interaction between them. In contrast, the spatiotemporal

interaction model includes an additional interaction term, allowing for unique variations in each region-period combination. S2 Fig depicts the marginal posterior mean of spatial, IID and temporal random effects with 95% credible intervals. The random effects analysis indicates that the IID random effect (neigh_id_index) has a stronger influence compared to the spatial random effect (denoted as i) and the temporal random effect (rw1_month_all), with the latter two exhibiting minimal variability. In the top panel, the spatial random effect remains close to zero across all observed SPDE mesh vertices, with very narrow credibility intervals. This suggests that the variability introduced by this random effect is minimal, and that it does not contribute significantly to the outcome. The middle panel displays the IID random effect for individual neighbourhood, representing individual-level variability. Here, substantial fluctuations are observed, with several indices showing large deviations from zero, accompanied by wider credibility intervals for specific sections of the index. This finding highlights the significant role that the IID random effect associated with neighbourhood factors plays in the model, contributing considerable variability to the outcome. The steep changes in mean values for certain neighbourhoods highlight strong spatial heterogeneity, suggesting that specific neighborhoods possess unique characteristics that drive differences in the outcome. In the bottom panel, the random effect for rw1_month_all, which captures temporal variation across months, remains relatively stable around zero. However, the wider credibility intervals, particularly at the extremes of the plot, suggest uncertainty in the estimation of this effect. This suggests that while there is some variability over time, the overall effect is small, and the wide intervals indicate uncertainty in how the model captures monthly fluctuations. The wider band towards the ends may reflect more uncertainty in the estimation for early and later months, which could result from less data or greater variability in those periods.

In contrast, S5 Fig depicts the random effects for the spatiotemporal interaction model. This figure contains four panels, with the final plot representing the spatiotemporal interaction effect. Notably, the IID random effect remains consistent across both models. However, the credible intervals for the spatial and temporal random effects are wider in the spatiotemporal interaction model, though the variation remains close to zero. This indicates increased uncertainty in these random effects estimates but suggests that their overall contribution remains minimal. In the last panel, the spatiotemporal interaction effect displays minimal variation, with values remaining relatively stable around zero. This stability indicates that the interaction effect contributes little additional variability to the model, suggesting limited region-period-specific deviations beyond those captured by the main spatial and temporal effects.

For the spatiotemporal separable model, the spatial effect parameters of κ and τ have mean values of 53.70 and 0.29, respectively. These values represent the marginal posterior distributions of the two hyperparameters for the spatial random field. Using these parameters, we calculated the spatial range r to be 0.452 km, or 452 meters. In contrast, for the spatiotemporal interaction model, we found of κ to be 35.62 and of τ to be 1.149, resulting in a spatial range of 0.064 km, or 64 meters. Notably, the spatiotemporal separable model shows a larger spatial range and higher κ value compared to the interaction model, indicating a broader influence of spatial correlations. To further assess the goodness-of-fit of our best-fitting model, we evaluated its predictive performance at unsampled locations [53]. Fig 4 reports the comparison between predicted COVID-19 infections derived from the best-fitting model and observed data. The Pearson correlation coefficient is 97%, which indicates a strong positive association between the predicted and the observed COVID-19 infections.

Fig 4. Comparison between the predicted and observed infection for the testing records

A dynamic map that depicts the prediction from the spatiotemporal interaction model has been developed in an R-shiny app and accessible through this link (covid19_Dakar). A sample view of the shiny app is presented in S6 Fig.

The risk of COVID-19 infection is higher in the western Dakar, particularly in the neighbourhoods of the following Communes: Fann-Point-E, Amitie, Plateau, Mermoz Sacre Coeur, Ngor, Sicap Liberte, Dieuppeul, Grand Yoff and Patte d'Oie. Central and Eastern peripheral areas have the lowest probabilities of COVID-19 infection.

4. Discussion

In this study, we developed Bayesian spatiotemporal models to examine the role of contextual risk factors in the spread of COVID-19 infection at the neighborhood level in Dakar. In addition to assessing the influence of these risk factors, we quantified the uncertainties associated with both the fixed effects of covariates and the random effects of spatial, temporal, and spatiotemporal components. A key outcome of this study is the mapping of areas at higher risk of COVID-19 infection in Dakar. Furthermore, the strong correlation between the predicted values from the model and the observed values underscores the high predictive accuracy of the best-fitted spatiotemporal interaction model.

To investigate the relationship between the covariates and COVID-19 infection, various contextual factors were considered. These factors were extracted from remote sensing images, which have proven useful for obtaining environmental and socioeconomic information. Remotely sensed socioeconomic and environmental variables have been widely applied to enhance our understanding of the local epidemiology of infectious diseases [54–59], including COVID-19 disease [60]. Population density is a very important feature of compact cities and an important proxy of socio-economic conditions along with land use characteristics such as built-up residential areas and built-up non-residential areas examined in this study. Regarding the contrasting statistical correlations that were previously found in the literature, showing a positive [10,14], then a negative correlation [5,8] between COVID-19 infection and population density, this study was an opportunity to further investigate this issue, by using very detailed data, and a more rigorous analytical approach. Our results confirm evidence of contrasting statistical correlations and highlight the impact of the scale of measurement on these correlations. On the one hand, COVID-19 infection was found to be significantly negatively associated with population density when no further adjustments were made and when population density was calculated using the total area of the spatial units as the denominator. On the other hand, when using adjusted population density, which considers only the built-up area as the denominator, a significant positive association with COVID-19 infection was observed. Therefore, adjusting population density before its application in our spatial statistical analysis has addressed issues related to the intrinsic properties of raw, non-adjusted population density such as the implicit assumption of uniformity over spatial units [13], the varying definitions of boundaries and the effect of varying scale of measurements [61]. Since the early stages of the pandemic, areas with high population density have experienced the greatest burden of COVID-19 infections [62,63]. Population density has significantly influenced the spatial distribution of COVID-19 [64], and has been a key indicator for implementing control measures such as physical distancing [65] that aimed to combat the spread of the disease. Our

findings link negative correlation between COVID-19 infection and population density to the omission of important methodological considerations. Conversely, the positive association that emerges following further adjustements confirms the role of population density as a primary risk factor for infectious diseases [61], including COVID-19 diseases.

Similarly, we found a significant positive association between residential areas and COVID-19 infection. In contrast, COVID-19 infection was negatively associated with non-residential areas, which include ACS areas. The distance to marketplaces was found to be not significant, suggesting that the effect of mobility was not fully captured by the geospatial predictors used in this study. Despite the complexities surrounding the influence of neighborhood features on the spread of COVID-19 [66], the positive association between COVID-19 infection and residential areas provides insights into local exposure in relation to built-up areas and land use characteristics. Additional information about residential dwellers, such as household social composition and income levels, could further reveal significant disparities in the spread of COVID-19 across residential areas [67].

The spatiotemporal model with space-time interaction demonstrated higher predictive performance compared to the non-spatial model and the spatiotemporal model with separable space-time effects. Including the space-time interaction improved the model performance as it accounts for specific local spikes, which cannot be explained by overall spatial or temporal effects alone. Indeed, the spatiotemporal interaction term has the benefit of capturing localised deviations in the infection risk, which is particularly valuable for identifying transient, area-specific surges, thereby enhancing model's sensitivity to local heterogeneity [40,41].

There is an important limitation that deserves to be highlighted. The epidemiological data provided by the Ministry of Health includes only the location of cases resulting from community transmission, therefore total case counts in some areas could be underrepresented. No information is available on the spatial distribution of other types of infection, such as the so-called "contact cases". Including these in the analysis could have refined our results. Furthermore, despite the low mortality rate from COVID-19, data on COVID-19 related-deaths could have provided additionnal insigths into the role of contextual risk factors in the spread of the disease. In this study, we aggregated the number of confirmed infection by months, which in turn smooths the daily reporting noise. Nonetheless, missing data, under-reporting and uneven testing across neighbourhoods may still affect the results. For instance, areas with better testing access might report more cases, which could confound the association with contextual risk factors. Potential data biases may arise from incomplete reporting or spatial variations in the detection of the infection, although greater concentration of health facilities and shared testing infrastructure in Dakar may limit these differences. Lastly, it is worth mentioning that, although remotely-sensed variables are useful proxies that capture meaningful landscape characteristics, census or survey-based household-level crowding and socioeconomic variables are useful indicators that could further refine our spatiotemporal model, and improve our understanding on the influence of contextual risk factors on COVID-19 infection.

5. Conclusion

The COVID-19 related social, health and economic impact has been gradually lessened, thanks to various efforts notably the rapid deployment of various vaccines that prompted a high level of population immunity. But in the post pandemic era, the disease is still circulating and the end of the global health emergency has led to the emergence of a global health threat. The risk of COVID-19

infection, or re-infection persists, as new virus variants can emerge, causing new surges in cases and deaths.

From a public health perspective, the research findings gained from this study can be valuable resources for health systems in low- and middle-income countries, for geographically targeted interventions. These outputs can serve as support tools for the optimised distribution and less-labour intensive delivery of the COVID-19 vaccine. They can support disease surveillance system through the monitoring of the potential COVID-19 outbreaks. Testing or vaccination efforts should prioritize high-risk western neighbourhoods, or dense residential areas.

Furthermore, the model can be adapted for the study of other infectious diseases, including malaria, tuberculosis and other neglected tropical diseases. Here we can leverage the flexibility of Bayesian spatiotemporal models, which has gained more popularity due to their capacity to incorporate spatial and temporal dependencies, uncertainties and intricate interactions. Bayesian computing has long been a complex task, but in the era of big data and the rapid advancement of computer technology, the Bayesian framework has been enhanced enough to allow in-depth investigations of epidemiological data, and the study of different diseases with a different and more extensive set of regressors.

Acknowledgment

This work was supported, in whole or in part, by the Gates Foundation under the GRID3 project. The conclusions and opinions expressed in this work are those of the author(s) alone and shall not be attributed to the Foundation. Under the grant conditions of the Foundation, a Creative Commons Attribution 4.0 License has already been assigned to the Author Accepted Manuscript version that might arise from this submission. Please note works submitted as a preprint have not undergone a peer review process.

The authors gratefully thanks the ASSESS project, funded by the ARES-CDD (https://www.ares-ac.be/fr), which provided the access to the neighbourhood's shapefile used as base layer to produce Fig 1, and 2 as well as S1 Fig and S6 Fig.

Data availability

The epidemiological data used in this study is publicly available through a request from the Ministry of Health. The geospatial predictors that we utilised to perform the analyses are freely available through the WorldpPop (https://www.worldpop.org/datacatalog/) and MAUPP data repositories (https://maupp.ulb.ac.be/page/grippa2018/). The neighbourhood shapefile used as base layer is accessible upon request through the following repository (distributed under CC-BY license): https://github.com/assanenian/INLA_Bayesian_Spatiotemporal

Code availability

All the code used to support this research, as well as the R-shiny app we developed to visualise the results are available in the following repository:

https://github.com/assanenian/INLA_Bayesian_Spatiotemporal

Supporting information

S1 Fig. SPDE triangulation of the study area

S2 Fig. Marginal posterior distributions of covariate coefficients for the separable spatio-temporal model

S3 Fig. Marginal posterior distributions of covariate coefficients for the spatiotemporal interaction model

S4 Fig. Marginal posterior distribution of the random effects components for the separable spatiotemporal model. Top: Marginal posterior mean of the spatial random effect $S(.)$
Middle: Marginal posterior mean of the iid effect $\mathbf{u_i}$
Bottom: Marginal posterior mean of the temporal random effect $\boldsymbol{\gamma_t}$

S5 Fig. ***Marginal posterior distribution of the random effects components for the spatiotemporal interaction model. Top panel: Marginal posterior mean of the spatial random effect $S(.)$***
Second panel: Marginal posterior mean of the iid effect u_i
Third panel: Marginal posterior mean of the temporal random effect γ_t
Bottom panel: Spatiotemporal interaction effect

S6 Fig. Overview of the R-Shiny App that visualizes the comparison between the observed and predicted COVID-19 prevalence.

References

1. Coronavirus [Internet]. [cited 2025 Apr 2]. Available from: https://www.who.int/health-topics/coronavirus

2. Mathieu E, Ritchie H, Rodés-Guirao L, Appel C, Gavrilov D, Giattino C, et al. Coronavirus (COVID-19) Vaccinations. Our World in Data [Internet]. 2024 Nov 15 [cited 2024 Nov 22]; Available from: https://ourworldindata.org/covid-vaccinations

3. Ndwandwe D, Wiysonge CS. COVID-19 vaccines. Current Opinion in Immunology. 2021 Aug 1;71:111–6.

4. Cohen C, Pulliam J. COVID-19 infection, reinfection, and the transition to endemicity. The Lancet. 2023 Mar 11;401(10379):798–800.

5. Carozzi F, Provenzano S, Roth S. Urban Density and Covid-19 [Internet]. Rochester, NY: Social Science Research Network; 2020 [cited 2025 May 7]. Available from: https://papers.ssrn.com/abstract=3643204

6. Cleary E, Atuhaire F, Sorichetta A, Ruktanonchai N, Ruktanonchai C, Cunningham A, et al. Comparing lagged impacts of mobility changes and environmental factors on COVID-19 waves in rural and urban India: A Bayesian spatiotemporal modelling study. PLOS Global Public Health. 2025 Apr 30;5(4):e0003431.

7. Cordes J, Castro MC. Spatial analysis of COVID-19 clusters and contextual factors in New York City. Spatial and Spatio-temporal Epidemiology. 2020 Aug 1;34:100355.

8. Hamidi S, Sabouri ,Sadegh, and Ewing R. Does Density Aggravate the COVID-19 Pandemic?: Early Findings and Lessons for Planners. Journal of the American Planning Association. 2020 Oct 1;86(4):495–509.

9. Huang H, Shi H, Zordan M, Lo SM, Tsou JY. Investigating the Spatiotemporal Relationship between the Built Environment and COVID-19 Transmission. ISPRS International Journal of Geo-Information. 2023;12(10).

10. Kadi N, Khelfaoui M. Population density, a factor in the spread of COVID-19 in Algeria: statistic study. Bull Natl Res Cent. 2020;44(1):138.

11. Rojas-Quiroz J, Marmolejo-Duarte C. Determining Equality of Infection Rates: A Spatial Analysis of Factors Associated with the Spread of COVID-19 in Barcelona, Spain. Journal of Urban Planning and Development. 2022;148(3).

12. Boyce MR, Katz R, Standley CJ. Risk Factors for Infectious Diseases in Urban Environments of Sub-Saharan Africa: A Systematic Review and Critical Appraisal of Evidence. Tropical Medicine and Infectious Disease. 2019 Dec;4(4):123.

13. Bhadra A, Mukherjee A, Sarkar K. Impact of population density on Covid-19 infected and mortality rate in India. Model Earth Syst Environ. 2021 Mar 1;7(1):623–9.

14. Garland P, Babbitt D, Bondarenko M, Sorichetta A, Tatem AJ, Johnson O. The COVID-19 pandemic as experienced by the individual [Internet]. arXiv; 2020 [cited 2025 May 7]. Available from: http://arxiv.org/abs/2005.01167

15. Oztig LI, Askin OE. Human mobility and coronavirus disease 2019 (COVID-19): a negative binomial regression analysis. Public Health. 2020 Aug;185:364–7.

16. Lai S, Ruktanonchai NW, Zhou L, Prosper O, Luo W, Floyd JR, et al. Effect of non-pharmaceutical interventions to contain COVID-19 in China. Nature. 2020 Sep;585(7825):410–3.

17. Lai S, Ruktanonchai NW, Carioli A, Ruktanonchai CW, Floyd JR, Prosper O, et al. Assessing the Effect of Global Travel and Contact Restrictions on Mitigating the COVID-19 Pandemic. Engineering. 2021 Jul 1;7(7):914–23.

18. Lai S, Bogoch II, Ruktanonchai NW, Watts A, Lu X, Yang W, et al. Assessing spread risk of COVID-19 in early 2020. Data Science and Management. 2022 Dec 1;5(4):212–8.

19. López-Arellano O, Bañuelos-Moreno M, Moreno-Altamirano A, López-Moreno S, Garrido-Latorre F. Socio-territorial inequalities and Covid-19 mortality in Mexico City. Salud Publica de Mexico. 2024;66(5):699–707.

20. Sun F, Matthews SA, Yang TC, Hu MH. A spatial analysis of the COVID-19 period prevalence in U.S. counties through June 28, 2020: where geography matters? Ann Epidemiol. 2020 Dec;52:54-59.e1.

21. Vandentorren S, Smaïli S, Chatignoux E, Maurel M, Alleaume C, Neufcourt L, et al. The effect of social deprivation on the dynamic of SARS-CoV-2 infection in France: a population-based analysis. The Lancet Public Health. 2022;7(3):e240–9.

22. Nazia N, Butt ZA, Bedard ML, Tang WC, Sehar H, Law J. Methods Used in the Spatial and Spatiotemporal Analysis of COVID-19 Epidemiology: A Systematic Review. International Journal of Environmental Research and Public Health. 2022 Jan;19(14):8267.

23. Chen D. Modeling the Spread of Infectious Diseases: A Review. In: Analyzing and Modeling Spatial and Temporal Dynamics of Infectious Diseases [Internet]. John Wiley & Sons, Ltd; 2014 [cited 2024 Dec 1]. p. 19–42. Available from: https://onlinelibrary.wiley.com/doi/abs/10.1002/9781118630013.ch2

24. Niraula P, Mateu J, Chaudhuri S. A Bayesian machine learning approach for spatio-temporal prediction of COVID-19 cases. Stoch Environ Res Risk Assess. 2022 Aug 1;36(8):2265–83.

25. Actualites | MINISTÈRE DE LA SANTÉ ET DE L'ACTION SOCIALE [Internet]. [cited 2025 Apr 7]. Available from: https://www.sante.gouv.sn/actualites

26. Petit V, Robin N, Martin N. Spatialité et temporalité de l'épidémie de la Covid-19 au Sénégal. Le processus de production des données sanitaires au regard des discontinuités territoriales. Revue francophone sur la santé et les territoires [Internet]. 2021 Jun 17 [cited 2024 Oct 24]; Available from: https://journals.openedition.org/rfst/1150

27. Tizzoni M, Bajardi P, Decuyper A, King GKK, Schneider CM, Blondel V, et al. On the Use of Human Mobility Proxies for Modeling Epidemics. PLOS Computational Biology. 2014 Jul 10;10(7):e1003716.

28. Gadiaga AN, De Longueville F, Georganos S, Grippa T, Dujardin S, Diène AN, et al. Neighbourhood-level housing quality indices for health assessment in Dakar, Senegal. Geospat Health [Internet]. 2021 May 5 [cited 2023 Jul 7];16(1). Available from: https://www.geospatialhealth.net/index.php/gh/article/view/910

29. Borderon M, Oliveau S, Machault V, Vignolles C, Lacaux JP, N'Donky A. Qualifier les espaces urbains à Dakar, Sénégal. Cybergeo: European Journal of Geography [Internet]. 2014 Mar 27 [cited 2025 Mar 18]; Available from: https://journals.openedition.org/cybergeo/26250

30. Agence Nationale de la Statistique et de la Démographie (ANSD) du Sénégal | «Un centre d'excellence dans un Système statistique national fort» [Internet]. [cited 2025 Apr 9]. Available from: https://www.ansd.sn/

31. Tais Grippa, Stefanos Georganos. Dakar very-high resolution land cover map [Internet]. Zenodo; 2018 [cited 2018 Oct 9]. Available from: https://zenodo.org/record/1290800#.W7xWQ_a2w2w

32. Grippa T, Georganos S. Dakar Land Use Map At Street Block Level [Internet]. Zenodo; 2018 [cited 2018 Oct 8]. Available from: https://zenodo.org/record/1291389

33. Georganos S, Gadiaga AN, Linard C, Grippa T, Vanhuysse S, Mboga N, et al. Modelling the Wealth Index of Demographic and Health Surveys within Cities Using Very High-Resolution Remotely Sensed Information. Remote Sensing. 2019 Jan;11(21):2543.

34. Grippa T, Linard C, Lennert M, Georganos S, Mboga N, Vanhuysse S, et al. Improving Urban Population Distribution Models with Very-High Resolution Satellite Information. Data. 2019 Mar;4(1):13.

35. Lloyd CT, Chamberlain H, Kerr D, Yetman G, Pistolesi L, Stevens FR, et al. Global spatio-temporally harmonised datasets for producing high-resolution gridded population distribution datasets. Big Earth Data. 2019;3(2):108–39.

36. Marconcini M, Metz- Marconcini A, Esch T, Gorelick N. Understanding Current Trends in Global Urbanisation - The World Settlement Footprint Suite. giforum. 2021;1:33–8.

37. Akinwande MO, Dikko HG, Samson A. Variance Inflation Factor: As a Condition for the Inclusion of Suppressor Variable(s) in Regression Analysis. Open Journal of Statistics. 2015;05(07):754–67.

38. O'brien RM. A Caution Regarding Rules of Thumb for Variance Inflation Factors. Qual Quant. 2007 Oct 1;41(5):673–90.

39. Barnett AG, Dobson AJ. Analysing Seasonal Health Data [Internet]. Berlin, Heidelberg: Springer; 2010 [cited 2025 Oct 28]. (Statistics for Biology and Health). Available from: http://link.springer.com/10.1007/978-3-642-10748-1

40. Simpson D, Rue H, Riebler A, Martins TG, Sørbye SH. Penalising Model Component Complexity: A Principled, Practical Approach to Constructing Priors. Statistical Science. 2017 Feb;32(1):1–28.

41. Blangiardo M, Cameletti M, Baio G, Rue H. Spatial and spatio-temporal models with R-INLA. Spatial and Spatio-temporal Epidemiology. 2013 Dec 1;7:39–55.

42. Moraga P, Dean C, Inoue J, Morawiecki P, Noureen SR, Wang F. Bayesian spatial modelling of geostatistical data using INLA and SPDE methods: A case study predicting malaria risk in Mozambique. Spatial and Spatio-temporal Epidemiology. 2021 Nov 1;39:100440.

43. Rue H, Riebler A, Sørbye SH, Illian JB, Simpson DP, Lindgren FK. Bayesian Computing with INLA: A Review. Annual Review of Statistics and Its Application. 2017 Mar 7;4(Volume 4, 2017):395–421.

44. Lambert D. Zero-Inflated Poisson Regression, with an Application to Defects in Manufacturing. Technometrics. 1992;34(1):1–14.

45. Rose CE, Martin ,S. W., Wannemuehler ,K. A., and Plikaytis BD. On the Use of Zero-Inflated and Hurdle Models for Modeling Vaccine Adverse Event Count Data. Journal of Biopharmaceutical Statistics. 2006 Aug 1;16(4):463–81.

46. Chaudhuri S, Juan ,Pablo, and Mateu J. Spatio-temporal modeling of traffic accidents incidence on urban road networks based on an explicit network triangulation. Journal of Applied Statistics. 2023 Dec 10;50(16):3229–50.

47. Gilks WR. Markov Chain Monte Carlo. In: Encyclopedia of Biostatistics [Internet]. John Wiley & Sons, Ltd; 2005 [cited 2025 May 9]. Available from: https://onlinelibrary.wiley.com/doi/abs/10.1002/0470011815.b2a14021

48. Brooks S, Gelman A, Jones G, Meng XL, editors. Handbook of Markov Chain Monte Carlo. New York: Chapman and Hall/CRC; 2011. 619 p.

49. Rue H, Martino S, Chopin N. Approximate Bayesian Inference for Latent Gaussian models by using Integrated Nested Laplace Approximations. Journal of the Royal Statistical Society Series B: Statistical Methodology. 2009 Apr 1;71(2):319–92.

50. Lindgren F, Rue H, Lindström J. An Explicit Link between Gaussian Fields and Gaussian Markov Random Fields: The Stochastic Partial Differential Equation Approach. Journal of the Royal Statistical Society Series B: Statistical Methodology. 2011 Sep 1;73(4):423–98.

51. Krainski E, Gómez-Rubio V, Bakka H, Lenzi A, Castro-Camilo D, Simpson D, et al. Advanced Spatial Modeling with Stochastic Partial Differential Equations Using R and INLA. New York: Chapman and Hall/CRC; 2018. 298 p.

52. Matérn B. Spatial variation. 2nd ed. Berlin, New York: Springer-Verlag; 1986. 151 p.

53. Juan Verdoy P. Enhancing the SPDE modeling of spatial point processes with INLA, applied to wildfires. Choosing the best mesh for each database. Communications in Statistics - Simulation and Computation. 2021 Oct 3;50(10):2990–3030.

54. Beginner's Guide to Spatial, Temporal and Spatial-Temporal Ecological Data Analysis with R-INLA. Volume 1 [Internet]. [cited 2025 May 9]. Available from: https://www.highstat.com/index.php/books2?view=article&id=11&catid=18

55. Aswi A, Cramb SM, Moraga P, Mengersen K. Bayesian spatial and spatio-temporal approaches to modelling dengue fever: a systematic review. Epidemiol Infect. 2018 Oct 29;147:e33.

56. Cheng J, Bambrick H, Tong S, Su H, Xu Z, Hu W. Winter temperature and myocardial infarction in Brisbane, Australia: Spatial and temporal analyses. Science of the Total Environment [Internet]. 2020 May 1 [cited 2024 Nov 19];715(136860). Available from: http://www.scopus.com/inward/record.url?scp=85078777422&partnerID=8YFLogxK

57. Georganos S, Brousse O, Dujardin S, Linard C, Casey D, Milliones M, et al. Modelling and mapping the intra-urban spatial distribution of Plasmodium falciparum parasite rate using very-high-resolution satellite derived indicators. Int J Health Geogr. 2020 Sep 21;19(1):38.

58. Guégan JF, Vargas Campos CA, Chevillon C, Sylla A, Dogbe M, Fast KM, et al. Land-use and land-cover changes and emerging non-tuberculous and tuberculous infectious diseases in humans and animals: a mixed-methods review of research findings from global and spatio-temporal perspectives. Environ Res: Health. 2025 Feb;3(1):012003.

59. Myer MH, Johnston JM. Spatiotemporal Bayesian modeling of West Nile virus: Identifying risk of infection in mosquitoes with local-scale predictors. Science of The Total Environment. 2019 Feb 10;650:2818–29.

60. Samrat A, Purse BV, Vanak A, Chaudhary A, Uday G, Rahman M, et al. Producing context specific land cover and land use maps of human-modified tropical forest landscapes for infectious disease applications. Science of The Total Environment. 2024 Feb 20;912:168772.

61. Paez A, Lopez FA, Menezes T, Cavalcanti R, Pitta MG da R. A Spatio-Temporal Analysis of the Environmental Correlates of COVID-19 Incidence in Spain. Geographical Analysis. 2021;53(3):397–421.

62. Salem G. La santé dans la ville: géographie d'un petit espace dense : Pikine (Sénégal). KARTHALA Editions; 1998. 420 p.

63. Ribeiro HV, Sunahara AS, Sutton J, Perc M, Hanley QS. City size and the spreading of COVID-19 in Brazil. PLoS One. 2020;15(9):e0239699.

64. Stier AJ, Berman MG, Bettencourt LMA. Early pandemic COVID-19 case growth rates increase with city size. npj Urban Sustain. 2021 Jun 28;1(1):1–6.

65. Wong DWS, Li Y. Spreading of COVID-19: Density matters. PLOS ONE. 2020 Dec 23;15(12):e0242398.

66. Chamberlain HR, Lazar AN, Tatem AJ. High-resolution estimates of social distancing feasibility, mapped for urban areas in sub-Saharan Africa. Sci Data. 2022 Nov 18;9(1):711.

67. Zhang X, Sun Z, Ashcroft T, Dozier M, Ostrishko K, Krishan P, et al. Compact cities and the Covid-19 pandemic: Systematic review of the associations between transmission of Covid-19 or other respiratory viruses and population density or other features of neighbourhood design. Health & Place. 2022 Jul 1;76:102827.

68. Hong B, Bonczak BJ, Gupta A, Thorpe LE, Kontokosta CE. Exposure density and neighborhood disparities in COVID-19 infection risk. Proceedings of the National Academy of Sciences. 2021 Mar 30;118(13):e2021258118.

Author contributions

Conceptualisation, methodology: ANG, MWT, AND; formal analysis and validation: AND and SC; data curation: MWT; Writing and editing: ANG, SC, NS, CL, SL, EC, CCN, AL, AJT, OY; supervision: AND.

Competing interests

No competing interests to declare

Click here to access/download;Figure;Fig 1.jpg

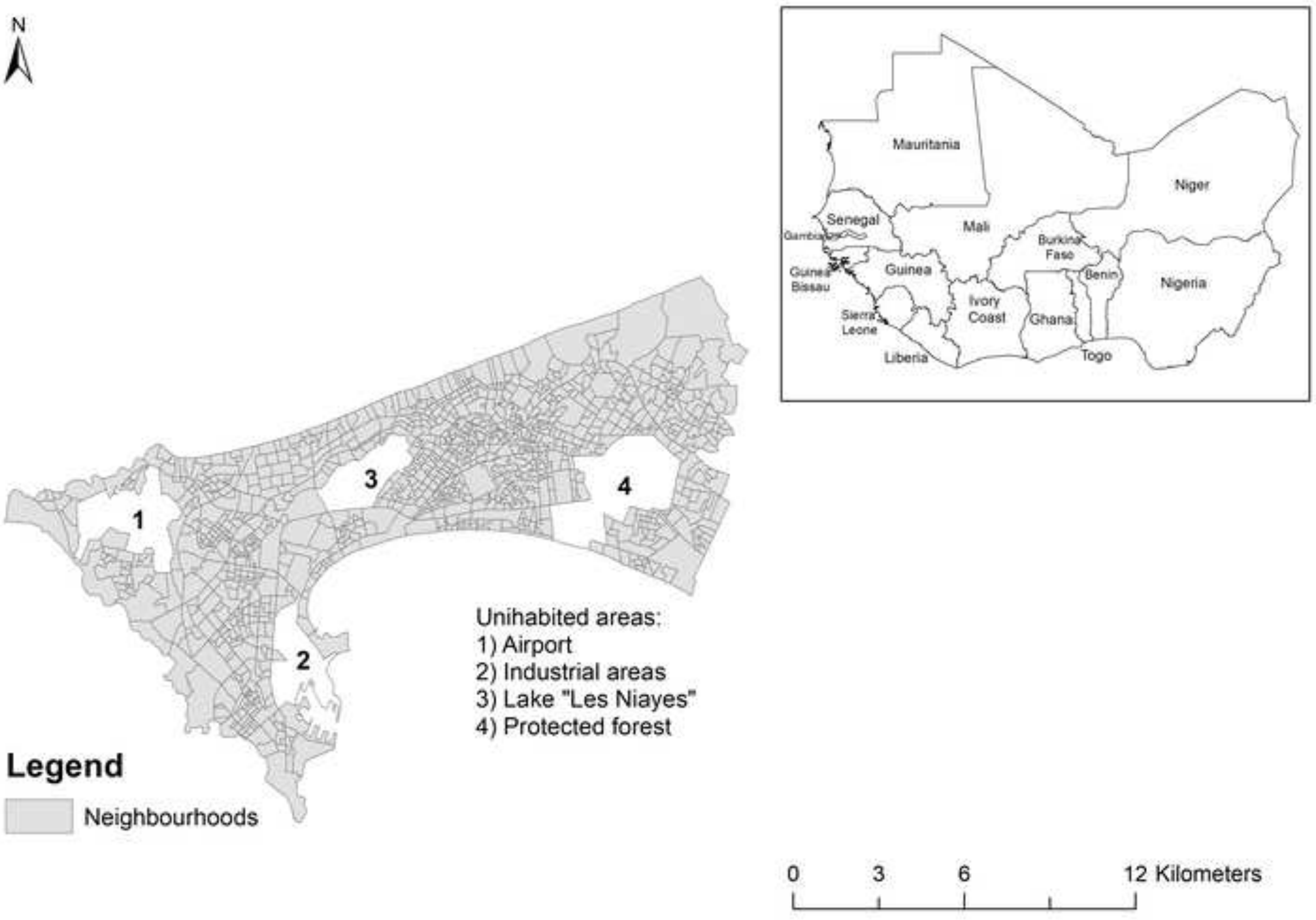

Click here to access/download;Figure;Fig 2.jpg

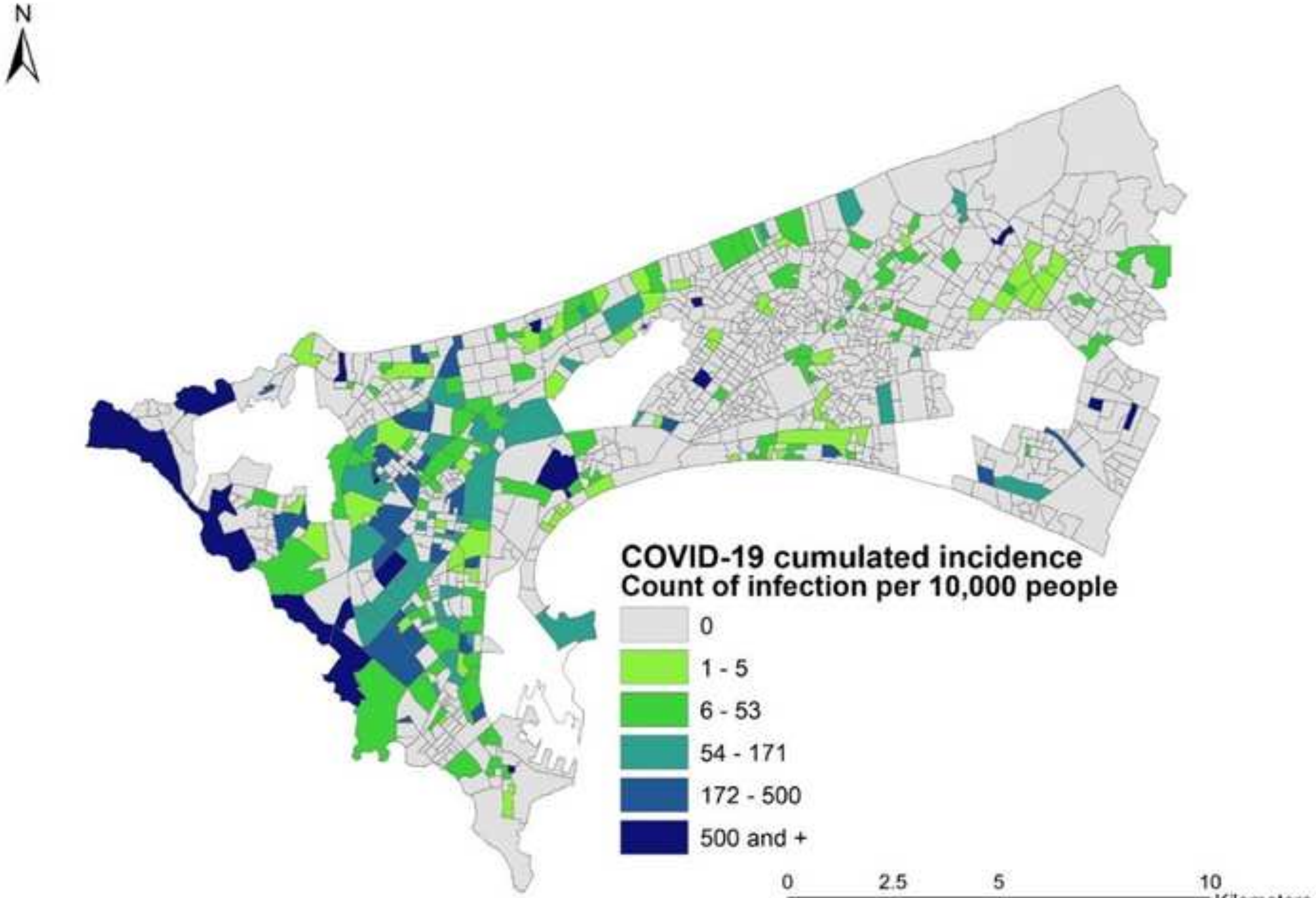

Figure

Click here to access/download;Figure;Fig 3.jpg

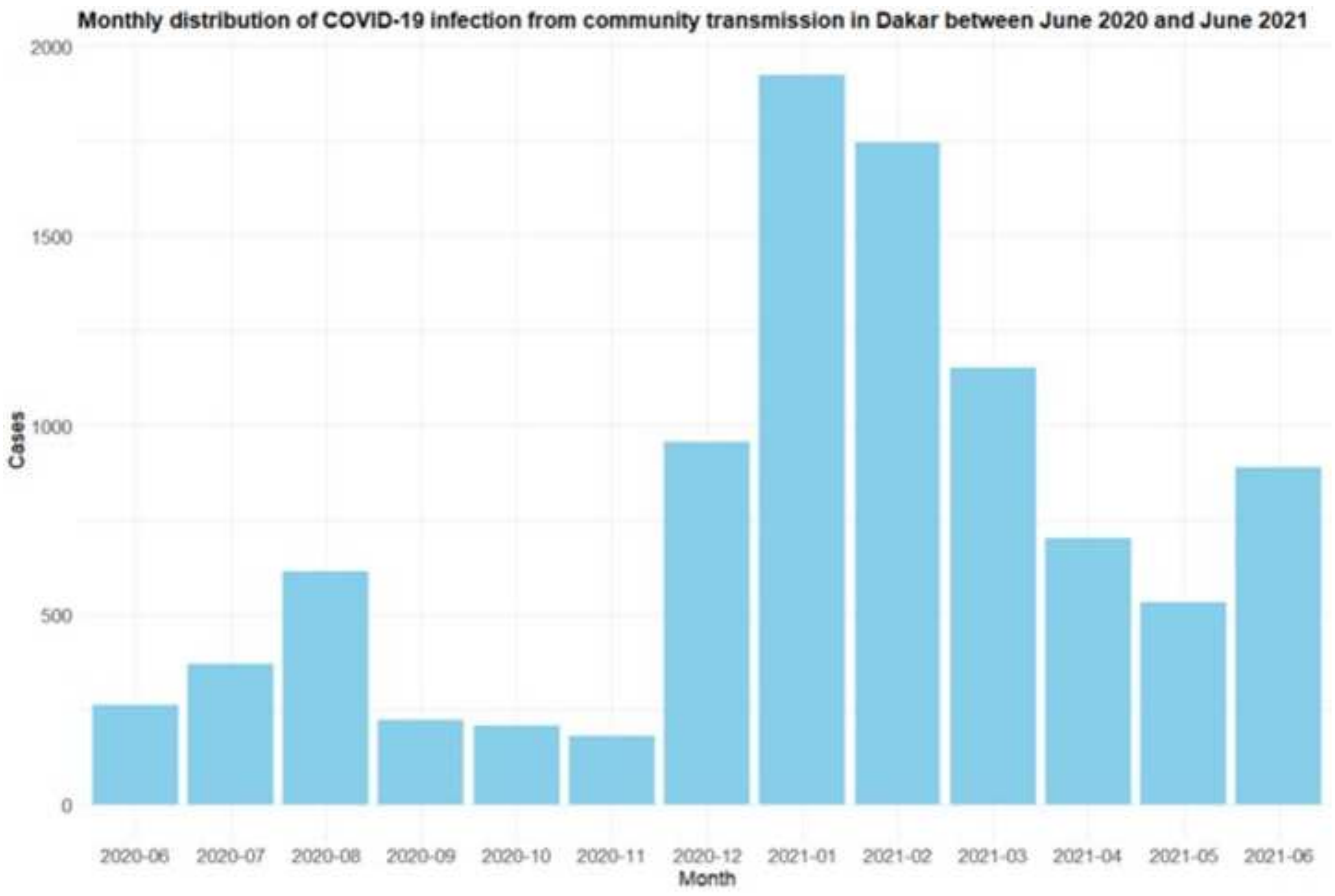

Figure

Click here to access/download;Figure;Fig 4.jpg

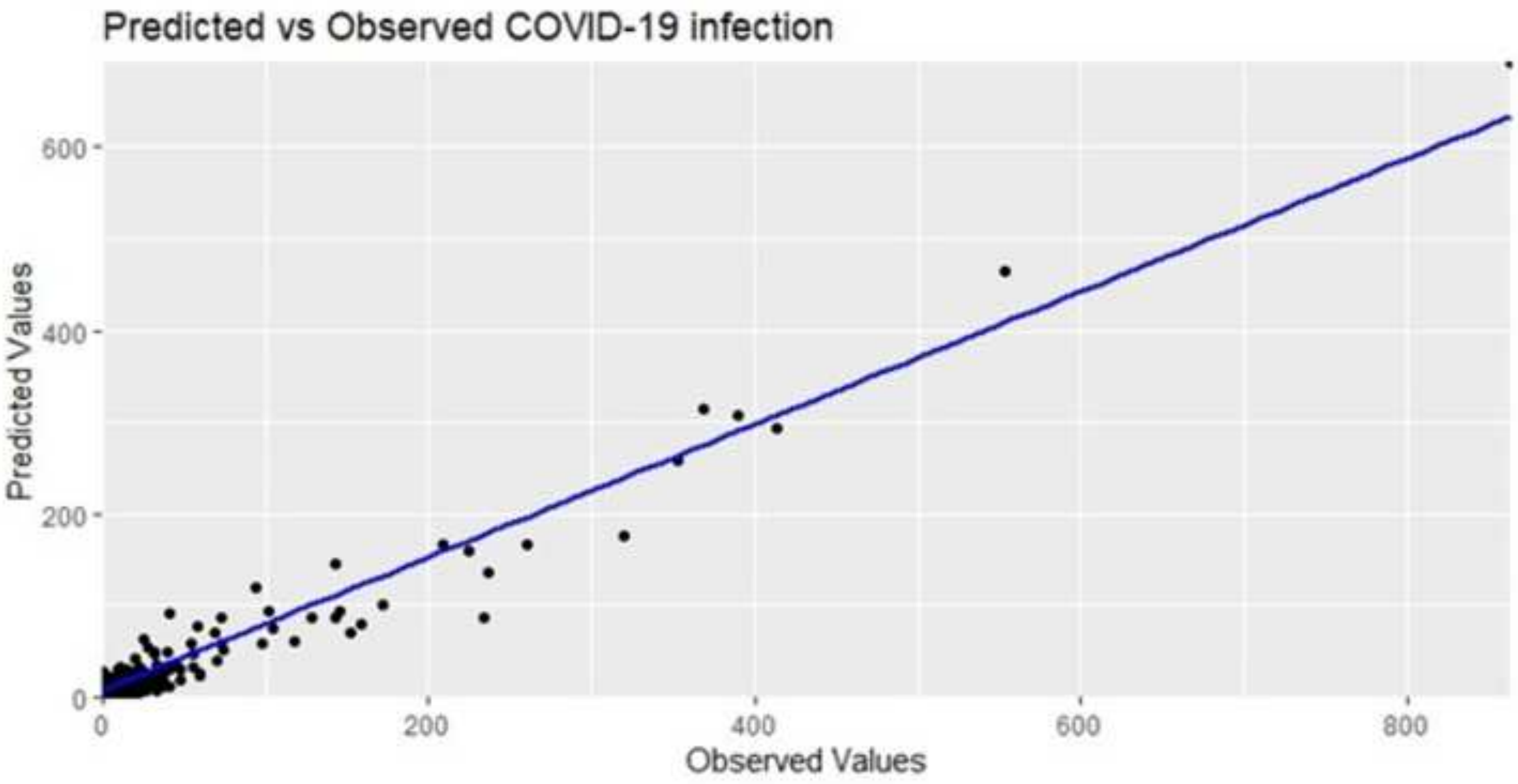

Click here to access/download
Supporting Information
S1 Fig.jpg

Click here to access/download
Supporting Information
S2 Fig.jpg

Click here to access/download
Supporting Information
S3 Fig.jpg

Click here to access/download
Supporting Information
S4 Fig.jpg

Click here to access/download
Supporting Information
S5 Fig.jpg

Click here to access/download
Supporting Information
S6 Fig.jpg

Spatio-temporal modelling of COVID-19 infection and associated risk factors in Dakar, Senegal

Authors and affiliation:

Assane N. Gadiaga[1,2*],, Mame Wodji Tine[2,3], Aminata Niang Diene[2,3], Catherine Linard[4], Niko Speybroeck[5], Ortis Yankey[1], Somnath Chaudhuri[1], Chibuzor Christopher Nnanatu[1], Eimear Cleary[1], Shengjie Lai[1], Attila N. Lazar[1], Andrew J. Tatem[1]

[1]WorldPop, Department of Geography and Environment, University of Southampton, Southampton, UK.

[2]Laboratoire dynamiques territoriales et sante, Université Cheikh Anta Diop de Dakar, Dakar, Senegal.

[3]Department of Geography, Université Cheikh Anta Diop de Dakar, Dakar, Senegal.

[4]Department of Geography, Université de Namur, Namur, Belgium.

[5]Université catholique de Louvain, Louvain-la-Neuve, Belgium.

[*]Corresponding author

Abstract

The spread of infectious diseases is a major threat to global health and economy and the recent COVID-19 pandemic is a perfect illustration of this. Appropriately modelling and accurate prediction of the outcome of disease spread over time and across space is a critical step towards informed development of effective strategies for public health interventions. In low and middle-income countries, however, the scarcity of spatially disaggregated time-series infectious diseases data often limits the analysis of the burden of infectious disease at a broad-scale, and the effects of the contextual risk factors is not often fully captured. In this study, we investigate the spatiotemporal patterns of COVID-19 infection in Dakar at the neighbourhood level, and evaluate the impact of potential risk factors. Geostatistical models based on COVID-19 infection were used to explain and predict the spatiotemporal distribution of COVID-19 infection between June 2020 and June 2021. We specified a Bayesian regression model that incorporates a spatio-temporally autocorrelated random effect in order to quantify the evolution of the spatial patterns of the COVID-19 infection overtime. Results show significant strong spatial heterogeneity but relatively small temporal variations of the COVID 19 distribution, and a positive association between adjusted population density (mean of the posterior probability: 0.29, credible interval: 0.24-0.34) and residential areas (mean of the posterior probability: 1.25, credible interval: 0.66-1.83) with COVID-19 infection. Western areas are at higher risk of COVID-19 infection compared to eastern and less densely populated peripheral neighbourhoods. Measuring the role of contextual risk factors and mapping the at-risk areas can provide valuable insights for policymakers in low- and middle-income countries, enabling more targeted public health interventions. These efforts also support the management of endemic diseases and preparedness for future outbreaks.

1. Introduction

The COVID-19 pandemic has had profound health, economic, and social consequences worldwide throughout the year 2020 until now. Since its emergence in late 2019, the disease has spread rapidly, leading to unprecedented public health responses such as lockdowns, social distancing measures, and mass vaccination campaigns [1]. As of April 2024, the COVID-19 related death toll had risen to more than 7 million from more than 700 million reported infections [2]. While widespread vaccination efforts have significantly reduced transmission and mortality [3], the containment of the virus is challenging because of the biological features of the virus [4], involving mutating variants that mitigate the effectiveness of the vaccine, leading to sustained rates of infection throughout the community.

Given the persistent risk of COVID-19 variants worldwide, assessing the disease spread and understanding its spatiotemporal patterns are key to effective monitoring, prevention and control. This is particularly true in many LMICs, where fragile disease surveillance system were unable to monitor the progress of COVID-19 and formulate appropriate responses. Recent studies have explored the role of various contextual factors in shaping the spread of COVID-19, including population density, landscape connectivity, human mobility, socioeconomic characteristics, environmental conditions, pollution and smoking [5–11]. Population density, in particular, has been widely investigated as a risk factor of disease transmission, given the fact that people's proximity to each other increases likelihood of infection [12]. In many cases, the relationship was positive, with more densely populated areas having higher rates of infections or experiencing early outbreaks [5,10,13,14]. Highly connected areas also face earlier and higher COVID 19 infection rates [15–18], and socioeconomic factors such as residential characteristics drive disparities in COVID 19 cases and deaths [7,19–21].

Several statistical approaches have been used to investigate the role of the aforementioned risk factors on the spread of the COVID-19 disease. They are either classical and frequentist methods, which remain at the core of most practical statistical applications [22], or Bayesian methods, which have been increasingly used in the field of spatial epidemiology due to their ability to incorporate spatial and temporal dependencies [23,24].

Although the scientific community is deploying efforts to understand contextual factors invloved in the spread of COVID-19, existing epidemiological data are not often resolved at spatial levels that allows for in-depth analyses; they are in most cases available at a large scale (i.e., regional or national levels) and the patterns of the disease at finer levels (i.e., at the intra urban levels) remains unknown.

In Senegal, the first case of COVID-19 was reported in early March 2020, and the majority of subsequent Senegalese cases were reported in Dakar, the capital city. According to the Ministry of Health, as of May 29, 2020, the Dakar region accounted for nearly three quarter (2525 cases out of the 3429 detected in the country; 73.6%) of reported COVID-19 infections in Senegal [25]. Public health authorities classified the source of the COVID-19 infection as one of the following four categories: (1) contact cases; (2) community cases; (3) imported cases and (4) evacuated cases. An infection was termed as a community case when the source of infection was unknown [26], making efforts to control the pandemic more challenging. Against this backdrop, the present study analysed the spatiotemporal dynamics of COVID-19 disease in Dakar, with a focus on the COVID-19 infection which arose from community transmission, and using COVID-19 time-series data at the finest administrative level.

Through a spatio-temporal Bayesian framework we evaluate the impact of population density, temperature, distance to marketplaces, non-pharmaceutical interventions such as ease of social distancing index, and residential characteristics on COVID-19 spread. Distance to marketplaces is used here as a proxy of mobility [27], while residential and non-residential areas indicates differences in living conditions and is a useful proxy on poverty levels [28]. In addition to the widely used population density (count of people per total area), adjusted population density that refers to the count of people per built-up area was also considered. This approach seeks to better capture the effect of crowding, because, although density expresses a ratio between population and an unit of area, these units are not uniformly distributed over the space. Our aim is to understand the spatiotemporal dynamic of COVID-19 spread in Dakar as well as related potential drivers. In a context of fragile health system, as in many low- and middle-income countries, having detailed time-series epidemiological data at the finest administrative level for Dakar offers a unique opportunity to explore advanced spatial statistical modelling approach and improve our understanding of localised transmission patterns. Understanding these localised transmission patterns is essential for designing targeted public health interventions, optimizing resource allocation, and mitigating the impact of future outbreaks.

2. Materials and methods

2.1. Study area and data

2.1.1. Study area

Dakar is the capital city of Senegal (Fig 1) and an example of African city that combines several aspects of the Africa's urban growth: rapid urbanization, land cover and land use changes, inequalities in living conditions [29] that have led to different health status [28]. Located in the westernmost part of the African continent (Fig 1), Dakar is the driving force of the Senegalese economy and through its markets, banks and industrial sector remains a magnet for migrants from all over the country. According to the last national census conducted in 2023, the region had a population of 4 million inhabitants, nearly a quarter of the Senegalese population (22 %) in an area representing only 0.3 % of the country [30]. The availability of georeferenced fine scale census information [28] and high quality, very-high resolution remote sensing land cover and land use products [31,32] has improved our knowledge on health inequalities and associated socioeconomic and environmental risk factors [28,33].

Fig 1. Geographical location of Dakar.

2.1.2. Data

The epidemiological data used in this study were obtained from the daily reports of COVID-19 infection in Dakar produced by the Ministry of health [25]. Data cover the period from 01/06/2020 to 30/06/2021 and records the number of confirmed COVID-19 infections from community-based transmission. Each anonymised record includes the date of the confirmed infection, the location of the infection and the number of new infections. There can be more than one record per date, with similar or different locations. The location of the infection often refers to the neighbourhood, which is the smallest administrative unit in an urban area. However, in some situations, the location can refer to a higher administrative extent, i.e. the commune, or a lower level spatial extent such as the housing unit. Geographic coordinates of each infection record were obtained by joining the excel-based disease data

file with the neighbourhood shapefile. To do this, the unique identifier availabe in the latter was assigned to the disease record when there is a matching between the location of the infection and the polygon shapefile. When the location of the infection was referring to a Commune or a housing unit, a fuzzy-matching algorithm was used to merge the infection record with the neighbourhood shapefile. After the geocoding of the infection records, the daily cases were aggregated by month for each neighbourhood. Thus, each neighbourhood has an aggregated count spanning the study period, providing an idea of the basic severity status of the neighborhood during the pandemic (covid19_Dakar). Although the neighborhood-level data offer an opportunity to examine the intra-urban variation of COVID-19 infection in Dakar, it is important to note that this does not provide a complete picture of the status of the pandemic in the city, as only infections from community transmission are analysed. In fact, only community based transmission can be used to reconstruct the spatio-temporal dynamic of the pandemic because, unlike the other type of infection, it includes information about the location of the infection (patient's residence).

Furthermore, just as there are multiple records for some dates, there are also days with no reported infections from community transmission. This might weaken the quality of the study, although it remains unclear whether the absence of reported infections is due to a lack of such cases among tested patients on the corresponding dates. The cumulative incidence and temporal distribution of COVID-19 infections over the study period are illustrated in Figs 2 and 3, respectively.

Fig 2. Aggregated incidence of COVID-19 confirmed infection from community transmission from June 2020 to June 2021.

Fig 3. Monthly distribution of COVID-19 infection from community transmission in Dakar from June 2020 to June 2021.

As predictors of COVID-19 infection, we used two high resolution satellite derived land use [32] and gridded population datasets [34], with a spatial resolution of 10m for the population data and street block level for the land use map. Additionally, distance to marketplaces, ease of social distancing index, and temperature data obtained from the WorldPop data repository were used [35]. The land use map depicts several spatial metrics, such as residential built-up areas (planned or unplanned), non-residential built-up areas (administrative, commercial and service areas), agricultural vegetation, natural vegetation, water bodies and baresoil areas. It is a precise representation of the Dakar's urban landscape, with an overall accuracy of 79 % [32] and regarding its usefulness in understanding inequalities in health and living conditions [28,33]. In the present study, however, this accuracy may be hampered by the mismatch between the year the satellite data were collected (2015) and the year in which the COVID-19 data were obtained (2020-2021). Although built and settlement layers are time-variant, we assume that this 5 year gap is negligible in the particular context of Dakar. To further assess this, we used the world settlement footprint data [36] and calculated the average percentage of change in the built-up areas at the neighbourhood level from 2015 to 2019. On average, built-up areas per neighbourhood have grown by 5% within the period 2015-2019 in Dakar, indicating minimal changes. It is important to note that the land use data does not cover Goree and Ngor islands as well as some parts of eastern Dakar. Thus, to ensure a full overlap between the COVID-19 data and the

geospatial predictors, the spatial extent of the study area was reduced and sparsely built-up areas in the eastern urbanisation front were excluded from the analysis.

Using these remotely sensed data sources, we first extracted the average residential built-up areas, non-residential built-up areas, temperature, and distance to marketplaces for each neighborhood. The total gridded population per neighborhood was divided by the neighborhood's total area to calculate population density. We then computed the adjusted population density by dividing the gridded population counts by the total built-up area for each neighborhood. Additionally, built-up area was divided by the neighborhood's total area to calculate built-up density. The covariates used for the modeling are listed in Table 1.

Table 1. List of covariates used for the modelling

Variable	Description	Source
**Temperature**	Annual average temperature	WorldPop
**Built-up density**	Built-up area per total area	MAUPP[a]
**Distance to marketplaces**	Average distance to marketplaces	WorldPop
**Population density**	Count of people per total area	MAUPP
**Adjusted population density**	Count of people per built-up area	MAUPP
**Residential areas**	Proportion of land use (LU) used for residential purposes	MAUPP
**Non-residential ACS areas**	Proportion of LU without residences used for administrative, commercial and services (ACS) purposes	MAUPP
**Ease of social distancing index**	Score index that evaluates the feasibility of social distancing measure	WorldPop

[a]MAUPP = Modelling and forecasting African Urban Population Patterns for vulnerability and heath assessments (https://maupp.ulb.ac.be/).

Statistical analyses were performed to check for multicollinearity among the covariates and to remove any covariates that were not statistically significant. To address this, we used the Variance Inflation Factor (VIF) to measure the level of collinearity. Covariates with a VIF greater than 5 were considered to have high multicollinearity and were removed from the analysis [37,38]. Only covariates with a VIF less than 5 and those that were statistically significant were selected for the final model. Among the ~~7~~ 8 variables considered for this analysis and introduced in Table 1, ~~only~~ built-up density and ease of social distancing index ~~was~~ were found showing multicollinearity issue and further dropped. In the following section, the Bayesian statistical modelling approach we developed to conduct this research is presented.

2.1.3. Ethics statements

The epidemiological datasets used in this study were completely anonymised and aggregated to administrative level to avoid confidentiality issues in accordance with relevant data protection regulations. The study adhered to the Declaration of Helsinki as it ensures respect for all participants

and protect their privacy, health and rights. Additionally, the data has been made publicly available in the official website of the Ministry of Health through a web-based visulisation platform.

2.2. Modelling COVID-19 infection risk

The spread of infectious diseases like COVID-19, involves complex patterns over both space and time. This complexity arises from the interactions between human behaviour, environmental factors, and disease transmission. To analyse COVID-19 cases in Dakar, Senegal, we have used a Bayesian spatiotemporal modelling approach. This method allows us to account for both spatial and temporal autocorrelation structures in the data. Spatial autocorrelation refers to the way disease cases are clustered in certain areas, while temporal autocorrelation deals with how cases change over time. Our modelling approach is important because COVID-19 cases are not evenly distributed across different locations or time periods, as illustrated in Figs 2 and 3.

In this study, we applied three distinct modelling approaches to analyse the spread of COVID-19 in Dakar. Our first approach focused on identifying monthly trends across the entire city. For this, we used both first-order and second-order random walk models (RW1 and RW2) to capture temporal randomness. To account for neighbourhood-level variations, we included an independent and identically distributed (IID) random effect. In this approach, we aggregated the total number of COVID-19 cases across Dakar for each month, using these monthly totals as the response variable.

In the second approach, we examined COVID-19 case counts at the neighbourhood level, capturing spatial variations within Dakar in addition to temporal trends. This separable spatiotemporal model included spatial randomness based on individual neighbourhoods, temporal randomness, and the IID effect of each neighbourhood. Finally, our third approach also involved spatiotemporal modelling, but with an added spatiotemporal interaction effect. This term represented an unobserved independent effect for each combination of neighbourhood and month, allowing us to capture unique patterns in each area over time. The rationale for constructing a temporal regression and spatiotemporal approaches is that temporal patterns often occurs in different aspects of health, particularly in infectious disease transmission [39]. Furthermore, infectious disease diffusion and spread is an intrinsically spatial process, as it involves complex interactions between human and different landscapes attributes. Therefore, the motivation for constructing multiple models in this study, and comparing them is that, in addition to temporal correlation, possible spatiotemporal correlation may play a crucial role, with the spatial correlation constant or changing over time.

2.2.1. Temporal modelling of COVID-19 infection

In the first approach, we have employed a temporal Poisson regression framework to analyse the temporal dynamics of COVID-19 in Dakar. In this model, the number of confirmed cases at each time point was represented using a Poisson likelihood in that we are dealing with a count data. The temporal resolution of the analysis has been set to one month, allowing us to explore trends and changes in the number of infected cases over time. Additionally, we incorporated geospatial variables to assess their potential correlations with the infection rates.

Let Y_t and E_t represent the number of observed and expected COVID-19 cases in the population at time t, where $t = 1, \ldots, T$. We assume that, given the relative risk q_t the number of observed cases follows a Poisson distribution:

$$Y_t \mid q_t \sim \text{Poisson}\ (\lambda_t = E_t\, q_t)$$

where E_t is the total population for the study area, and the log-risk is modelled as:

$$\log(q_t) = \beta_0 + Z_t^T \beta + u_i + \gamma_t \text{ ……….. (1)}$$

where, Z_t represents the matrix of covariates used to account for various factors influencing the infection rates. The term u_i is an IID random effect that captures unobserved variability across the neighbourhoods. Additionally, γ_t represents the temporal effects, which can be modelled using either a RW1 or a RW2 to account for the temporal structure and potential trends over time. In the case of the RW1, the temporal effects γ_t are modeled such that the increments $\Delta\gamma_t = \gamma_t - \gamma_{t-1}$ follow a Gaussian distribution with zero mean and precision τ. On the other hand, for a RW2, the temporal effects are modeled as $\Delta^2 \gamma_t = \gamma_t - 2\gamma_{t-1} + \gamma_{t-2}$ also follow a Gaussian distribution with zero mean and precision τ. This RW2 model provides a smoother temporal evolution over time compared to the RW1 model, making it particularly useful for capturing complex temporal patterns such as trends, cycles, or deviations from simple linear temporal patterns. For prior selection, we have used penalized complexity (PC) priors [39]. For the IID random effect, the PC prior on the precision was set to [0.5, 0.01], where 0.5 is the central tendency and 0.01 controls the spread or uncertainty around it. For the RW1 or RW2 model on the monthly data, the PC prior on the precision with parameters [1, 0.01] represents a location parameter of 1 and a scale parameter of 0.01, indicating a prior belief that the precision is centered around 1 with relatively low uncertainty. The prior for the coefficient vector $\beta = (\beta_0, \ldots, \beta_p)$ which follows a zero-mean Gaussian distribution with a precision of 0.001. All log-precision parameters followed inverse gamma distributions with shape parameter ($\alpha = 1$) and scale parameter ($\beta = 0.00005$).

2.2.2. Spatiotemporal modelling of COVID-19 infection

In the second approach, we employed a spatiotemporal Poisson regression framework to analyze the spread of COVID-19 in Dakar. In this model, the number of confirmed cases at each spatial unit (neighborhood) and time point was represented using a Poisson likelihood. Similar to the first approach, the temporal resolution of the spatiotemporal analysis was set to one month, allowing us to observe trends and changes in case numbers over time (months). The spatiotemporal model included spatial random effects to account for the influence of neighboring regions on case numbers. It also incorporated temporal random effects to capture trends and seasonal variations over time. Additionally, geospatial covariates were included to explore their potential associations with infection rates.

Let Y_{it} and E_{it} represent the number of observed and expected COVID-19 infected cases in the i-th area (neighbourhood) and the t-th period (month), where $t = 1, \ldots, T$. We assume that, given the relative risk q_{it} , the number of observed cases follows a Poisson distribution:

$$Y_{it} \mid q_{it} \sim \text{Poisson} (\lambda_{it} = E_{it}\, q_{it})$$

where, E_{it} are the total population for individual neighbourhood of the study area and the log-risk is modelled as in Equation (2) for spatiotemporal separable model and in Equation (3) for spatiotemporal interaction model.

$$\log(q_{it}) = \beta_0 + Z_{it}^T\beta_{it} + S(x_i) + u_i + \gamma_t \text{ .... (2)}$$

$$\log(q_{it}) = \beta_0 + Z_{it}^T\beta_{it} + S(x_i) + u_i + \gamma_t + \delta_{it} \text{ .... (3)}$$

where in both Equation (2) and (3), $S(.)$ represents the spatially structured random effects, and Z_{it} represents the matrix of covariates used in the model. The temporally structured effect has been represented by γ_t. In Equation (3), δ_{it} represents the spatio-temporal interaction between the two structured effects. The IID random effect u_i is added to the model to capture neighbourhood-level variability with a penalized complexity (PC) prior on the precision set as $[0.5, 0.01]$ where we assumed a prior mean of 0.5 and a prior precision of 0.01. The matrix of covariates was built by stacking the covariate values for every space-time combination, and separate random effect mapping matrices link these observations to spatial, temporal or spatiotemporal latent effects. We have modelled temporal trends using a RW1 for the monthly data. This model was scaled for identifiability, and we applied a PC prior on the precision with parameters [1,0.01] with a prior mean of 1 and a prior precision of 0.01. For all prior selections, we used PC priors following the framework proposed by Simpson et al. [39]. We have assigned a vague prior to the vector of coefficients $\beta = (\beta_0, \ldots, \beta_p)$ which follows a zero-mean Gaussian distribution with a precision of 0.001. Finally, all parameters associated with log-precisions are assigned inverse Gamma distributions with shape parameter $(\alpha = 1)$ and scale parameter $(\beta = 0.00005)$. The default prior distributions for all parameters in R-INLA were selected based on commonly used priors in previous studies [40–42]. Our findings demonstrate robustness to alternative priors. We have tested multiple scenarios with different priors and consistently obtained the same results. As shown in Equation (3), we introduced a spatiotemporal interaction effect as an unobserved, independent term for each region-period combination (i, t), modelled as $\delta_{it} \sim Normal\left(0, \frac{1}{\tau\delta}\right)$. This interaction term assumes no additional structure, representing independent variations from the main spatial and temporal effects when those are included in the model. It captures global space-time heterogeneity and is typically modeled as white noise, as discussed in previous studies [40]. This interaction term offers a more flexible structure, capturing localized deviations from the main spatial and temporal patterns. To compare modelling strategies, we constructed the third model set that incorporate all covariates, using both spatiotemporal interactions and PC priors. A summary of these competing models is presented in Table 2 in the Results Section.

The dataset used in this study shows zero inflation. As such, zero-inflated Poisson and Poisson Hurdle models can be used to accommodate zero-inflated discrete distributions. While ZIP models add extra probability mass to the zero outcome [43], Poisson hurdle models, on the other hand, are characterized by a mixture of zero and non-zero outcomes [44,45]. In a ZIP model, the response variable Y_{it} is zero with probability π, and follows a Poisson distribution with parameter λ_{it} with probability $1 - \pi$. In particular,

$$Y_{it} = \begin{cases} 0, & \text{with prob } \pi + (1-\pi)e^{-\lambda_{it}} \\ k, & \text{with prob } (1-\pi)\,\frac{\lambda_{it}^{k} e^{-\lambda_{it}}}{k!},\; k \geq 1 \end{cases} \quad \text{.........} (4)$$

Conversely, in a Poisson hurdle model, the response variable Y_{it} is zero with probability π, and follows a truncated Poisson distribution with parameter λ_{it} with probability $1-\pi$. Mathematically,

$$Y_{it} = \begin{cases} 0, & \text{with prob } \pi \\ k, & \text{with prob } \frac{(1-\pi)}{1-e^{-\lambda_{it}}}\left(\frac{\lambda_{it}^{k} e^{-\lambda_{it}}}{k!}\right),\; k \geq 1 \end{cases} \quad \text{.........} (5)$$

In the model fitting process, Bayesian inference typically employs Markov Chain Monte Carlo (MCMC) methods to estimate the joint posterior distribution of model parameters [46,47]. But for big dataset such as the time-series data used in this study, this can be computationally intensive. As a faster alternative, the integrated nested Laplace approximation (INLA) approximates posterior marginal distributions efficiently, especially for models represented as latent Gaussian Markov random fields (GMRF) [42,48]. In the current study, we have applied INLA along with a stochastic partial differential equation (SPDE) method. The SPDE method allows us to model a continuous spatial process, such as a Gaussian field (GF), using a discretely indexed spatial random process like a GMRF [49,50].

In particular, the spatial random process represented by $S(.)$ explicitly denote dependence on the spatial field, follows a zero-mean Gaussian process with Matérn covariance function [51] represented as:

$$\mathrm{Cov}\left(S(x_i), S(x_j)\right) = \frac{\sigma^2}{2^{\nu-1}\Gamma(\nu)}\left(\kappa \parallel x_i - x_j \parallel\right)^{\nu} K_{\nu}\left(\kappa \parallel x_i - x_j \parallel\right)$$

The parameter σ^2 represents the process variance, indicating the variability of the values around the mean. The smoothness parameter $\nu > 0$ influences the rate of decay of correlations with distance, while the scaling parameter $\kappa > 0$ governs the overall range of the covariance. The term $\parallel x_i - x_j \parallel$ denotes the Euclidean distance between locations. The modified Bessel function of the second kind, $K_{\nu}(.)$, defines the covariance structure, and $\Gamma(\nu)$ is the gamma function, normalizing the covariance for scaling purposes.

The parameterized model we follow is of the form:

$$(\kappa^2 - \Delta)^{(\alpha/2)}(\tau S) = W \text{ on } \mathbb{R}^d$$

where $\Delta = \sum_{i=1}^{d} \frac{\partial^2}{\partial x_i^2}$ is the Laplacian, $\alpha = (\nu + d/2)$ is the smoothness parameter, τ is inversely proportional to σ, W is Gaussian white noise and $\kappa > 0$ is the scale parameter, related to range r, defined as the distance at which the spatial correlation becomes negligible. For each ν, we have $r = \sqrt{8\nu}/\kappa$, with r corresponding to the distance where the spatial correlation is close to 0.1. Note that we have $d = 2$ for a two-dimensional process, and we fix $\nu = 1$, so that $\alpha = 2$ in this case [40–42]. In the current study, we have applied the PC priors [39] for the Matérn model, which helps prevent overfitting by supporting simpler models. The prior for the marginal standard deviation is set as [1,0.1] where we assumed a prior mean of 1 and a prior precision of 0.1. This allows some flexibility around a mean of 1 based on our expectations of spatial variation. We utilized the centroids of each neighborhood as the target locations for SPDE mesh construction. According to Verdoy [52], an optimal mesh should possess a sufficient number of vertices to facilitate effective predictions, and within a limit to have control over computational time. Following this principle, the triangulated mesh has been generated using inla.mesh.2d function from R-INLA package, based on Delaunay triangulation method [50], with the best fitting mesh having 7365 vertices for the 1078 neighbourhoods considered in this

study~~we selected the best-fitting mesh having 7365 vertices~~. S1 Fig depicts the SPDE mesh designed for the study area with 1078 neighborhood centroid locations highlighted as red points and the blue line marks the boundary of the study area. We have created a projection matrix to transfer the spatially continuous Gaussian random field from the observations to the mesh nodes. The centroids of the neighbourhoods and the triangulation in the mesh are used to develop this projection matrix.

All models assume that the number of COVID-19 cases follows a Poisson likelihood, with the expected count determined by population size and relative risk. The models differ in how spatial and temporal dependencies are incorporated. The temporal model includes monthly random effects modelled as either RW1 or RW2 processes, while the spatiotemporal models incorporate both spatially structured effects (using SPDE approach) and temporally structured effects (RW1). The interaction model further includes an independent spatiotemporal interaction term to capture unobserved localized deviations. Estimated parameters are inferred from the data through posterior distributions and include the regression coefficients (β), IID random effects (u_i), temporal effects (γ_t), spatiotemporal interactions (δ_{it}), and spatial field values ($S(x_i)$). Assumed parameters are chosen based on prior knowledge or literature, such as the parameters of the PC priors ([0.5, 0.01] for u_i, [1, 0.01] for γ_t) or the prior for the SPDE spatial range [40]. Fixed parameters are held constant and define model structure, such as the Matérn smoothness parameter ν and the spatial dimension [50]. A summary of estimated parameters and priors is presented in table 2.

Table 2. Estimated parameters for the spatiotemporal models.

**Parameter**	**Description**	**Model(s) Used**	**Prior Distribution**
β_0, β	Fixed effects (intercept, covariates)	All models	Normal(0, 0.001)
u_i	IID random effect (neighbourhood-level variability)	Spatiotemporal	PC prior [0.5, 0.01]
γ_t	Temporal random effect (RW1 or RW2)	Temporal, Spatiotemporal	PC prior [1, 0.01]
δ_{it}	Spatiotemporal interaction term	Interaction model only	Normal(0, 1/$\tau\delta$)
$S(x_i)$	Spatially structured effect (SPDE)	Spatiotemporal	Matérn covariance, PC prior [1, 0.1]
τ	Precision parameters (for random effects)	All models	Inverse Gamma(1, 0.00005)
ν	Smoothness parameter in SPDE	Spatiotemporal	Fixed at 1
κ	Scale parameter in SPDE	Spatiotemporal	Derived from range r

In the model fitting process, we have included ~~seven~~ eight covariates, such as population density, adjusted population density, built-up density, average value of temperature, residential built-up areas, non-residential built-up areas, distance to marketplaces, ease of social distancing index, as outlined in Section 2.1. The variable month spans from 1 to 12, representing each month of the year, and month is treated as factor in the model. We used data on COVID-19 cases from June 2020 to May 2021 (a total of 12 months) to fit the model described in Equation (2). To assess prediction accuracy, we held out data from the following month, June 2021, as a test set. Using this fitted model, INLA then predicted the COVID-19 cases for these held-out months by computing the predictive distributions based on the posterior distributions of the model parameters and latent variables. This approach allows INLA to provide both point predictions and uncertainty estimates, making it suitable for evaluating model accuracy on out of sample data. Finally, we compared the predicted values with the original observed COVID-19 cases from June 2021 to evaluate the predictive performance of the model, with detailed comparison results and performance metrics discussed in Section 3. All the analyses were conducted in R statistical software (v.4.2.2).

3. Results

The following results summarize the outcomes of our Bayesian spatiotemporal modelling approaches, highlighting the temporal dynamics, spatial heterogeneity, and zero-inflation characteristics of COVID-19 case distributions in Dakar. For the latter, a ZIP hurdle distribution is used in the modelling, and the parameter significance as well as the goodness of fit of the three fitted Bayesian ZIP hurdle models are presented in Table ~~2~~3. It appears that the spatiotemporal interaction model was the best model to fit the data. Indeed, the best fitting spatiotemporal interaction model (M3) has the lowest Deviance Information Criterion (DIC) and Watanabe-Akaike information Criterion (WAIC) values compared to the temporal (M1) and separable spatiotemporal model (M2). The DIC and WAIC values varied slightly from the temporal to the separable spatial-temporal models (37050 and 37049 DIC values for M1 and M2, respectively, then 37061 and 37060 WAIC values for M1 and M2, respectively). When a spatiotemporal interaction is specified, the DIC and WAIC has significantly dropped, indicating higher predictability when the space-time interaction is accounted for.

Table ~~2~~3. Posterior estimates of model parameters of temporal and spatiotemporal models of COVID-19 in Dakar.

			**Spatiotemporal Model**			
	**Temporal Model (M1)**		**Separable Model (M2)**		**Interaction model (M3)**	
**Variable**	**Mean**	**95% BCI [a]**	**Mean**	**95% BCI**	**Mean**	**95% BCI**
**Intercept**	-10.23	-55.38,-34.91	-10.25	-55.42,34.92	-10.27	-55.46,34.92
**Residential areas**	1.25	0.67,1.83	1.25	0.67,1.83	1.25	0.66,1.83

**Non-residential areas**	-0.66	-1.29,-0.04	-0.66	-1.29,-0.04	-0.67	-1.29,-0.04
**Population density**	-0.003	-0.003,-0.002	-0.003	-0.003,-0.002	-0.003	-0.003,-0.002
**Adjusted population density**	0.29	0.24,0.34	0.29	0.24,0.34	0.29	0.24,0.34
**Temperature**	0.03	-0.12,-0.18	0.03	-0.12,0.18	0.03	-0.12,0.18
**Distance to marketplaces**	0.00	0.00,0.00	0.00	0.00,0.00	0.00	0.00,0.00
**DIC [b]**	37044		37043		36711	
**WAIC [c]**	37056		37055		36885	

[a] BCI: Bayesian Credible Interval

[b] DIC: Deviance Information Criterion

[c] WAIC: Watanabe-Akaike Information Criterion

For the models specified in Equations (2) and (3), we examine the posterior distributions of fixed and random effects for both the separable spatiotemporal model and the spatiotemporal interaction model. These posterior distributions are presented in S2 and S3 Figs, and S4 and S5 Figs. For both models, the posterior distribution of the fixed effects provides insights into the impact of covariates on the outcome across space and time.

The results indicate that the covariates month number, distance to marketplace, and temperature are non-significant in both models, with null posterior mean or zero included within the range of the 95% credible interval. Conversely, population density and non-residential ACS areas show a significant negative effect, suggesting a negative association in the model. As the proportion of non-residential areas, and the count of people per hectare per neighbourhood's total area increase, the expected mean of the COVID-19 infection decrease by 0.003 and 0.67, respectively. These expected values fall within the 95% credible interval for both the effect of non-residential areas (-1.29, -0.04) and population density (-0.003, -0.002). Adjusted population density and residential areas exhibit a significant positive effect, indicating a strong positive influence. When the proportion of residential areas, and the count of people per built-up area increase, the expected mean of COVID-19 infection increase by 0.29 and 1.25, respectively. Also there is a 95% probability this effect lies between 0.66 and 1.83 for residential areas, and between 0.24 and 0.34 for adjusted population density. For both spatiotemporal models, the fixed effects demonstrate a similar trend and nature.

On the other hand, the posterior distribution of the random effects captures underlying spatial, temporal, and spatiotemporal variability. In the separable model, spatial and temporal effects are modeled independently, assuming no interaction between them. In contrast, the spatiotemporal

interaction model includes an additional interaction term, allowing for unique variations in each region-period combination. S2 Fig depicts the marginal posterior mean of spatial, IID and temporal random effects with 95% credible intervals. The random effects analysis indicates that the IID random effect (neigh_id_index) has a stronger influence compared to the spatial random effect (denoted as i) and the temporal random effect (rw1_month_all), with the latter two exhibiting minimal variability. In the top panel, the spatial random effect remains close to zero across all observed SPDE mesh vertices, with very narrow credibility intervals. This suggests that the variability introduced by this random effect is minimal, and that it does not contribute significantly to the outcome. The middle panel displays the IID random effect for individual neighbourhood, representing individual-level variability. Here, substantial fluctuations are observed, with several indices showing large deviations from zero, accompanied by wider credibility intervals for specific sections of the index. This finding highlights the significant role that the IID random effect associated with neighbourhood factors plays in the model, contributing considerable variability to the outcome. The steep changes in mean values for certain neighbourhoods highlight strong spatial heterogeneity, suggesting that specific neighborhoods possess unique characteristics that drive differences in the outcome. In the bottom panel, the random effect for rw1_month_all, which captures temporal variation across months, remains relatively stable around zero. However, the wider credibility intervals, particularly at the extremes of the plot, suggest uncertainty in the estimation of this effect. This suggests that while there is some variability over time, the overall effect is small, and the wide intervals indicate uncertainty in how the model captures monthly fluctuations. The wider band towards the ends may reflect more uncertainty in the estimation for early and later months, which could result from less data or greater variability in those periods.

In contrast, S5 Fig depicts the random effects for the spatiotemporal interaction model. This figure contains four panels, with the final plot representing the spatiotemporal interaction effect. Notably, the IID random effect remains consistent across both models. However, the credible intervals for the spatial and temporal random effects are wider in the spatiotemporal interaction model, though the variation remains close to zero. This indicates increased uncertainty in these random effects estimates but suggests that their overall contribution remains minimal. In the last panel, the spatiotemporal interaction effect displays minimal variation, with values remaining relatively stable around zero. This stability indicates that the interaction effect contributes little additional variability to the model, suggesting limited region-period-specific deviations beyond those captured by the main spatial and temporal effects.

For the spatiotemporal separable model, the spatial effect parameters of κ and τ have mean values of 53.70 and 0.29, respectively. These values represent the marginal posterior distributions of the two hyperparameters for the spatial random field. Using these parameters, we calculated the spatial range r to be 0.452 km, or 452 meters. In contrast, for the spatiotemporal interaction model, we found of κ to be 35.62 and of τ to be 1.149, resulting in a spatial range of 0.064 km, or 64 meters. Notably, the spatiotemporal separable model shows a larger spatial range and higher κ value compared to the interaction model, indicating a broader influence of spatial correlations. To further assess the goodness-of-fit of our best-fitting model, we evaluated its predictive performance at unsampled locations [53]. Fig 4 reports the comparison between predicted COVID-19 infections derived from the best-fitting model and observed data~~COVID-19 infection~~. The ~~pearson~~ Pearson correlation coefficient is 97%, which indicates a strong positive association between the predicted and the observed COVID-19 infections.

Fig 4. Comparison between the predicted and observed infection for the testing records

A dynamic map that depicts the prediction from the spatiotemporal interaction model has been developed in an R-shiny app and accessible through this link (covid19_Dakar). A sample view of the shiny app is presented in S6 Fig.

The risk of COVID-19 infection is higher in the western Dakar, particularly in the neighbourhoods of the following Communes: Fann-Point-E, Amitie, Plateau, Mermoz Sacre Coeur, Ngor, Sicap Liberte, Dieuppeul, Grand Yoff and Patte d'Oie. Central and Eastern peripheral areas have the lowest probabilities of COVID-19 infection.

4. Discussion

In this study, we developed Bayesian spatiotemporal models to examine the role of contextual risk factors in the spread of COVID-19 infection at the neighborhood level in Dakar. In addition to assessing the influence of these risk factors, we quantified the uncertainties associated with both the fixed effects of covariates and the random effects of spatial, temporal, and spatiotemporal components. A key outcome of this study is the mapping of areas at higher risk of COVID-19 infection in Dakar. Furthermore, the strong correlation between the predicted values from the model and the observed values underscores the high predictive accuracy of the best-fitted spatiotemporal interaction model.

To investigate the relationship between the covariates and COVID-19 infection, various contextual factors were considered. These factors were extracted from remote sensing images, which have proven useful for obtaining environmental and socioeconomic information. Remotely sensed socioeconomic and environmental variables have been widely applied to enhance our understanding of the local epidemiology of infectious diseases [54–59], including COVID-19 disease [60]. Population density is a very important feature of compact cities and an important proxy of socio-economic conditions along with land use characteristics such as built-up residential areas and built-up non-residential areas examined in this study. Regarding the contrasting statistical correlations that were previously found in the literature, showing a positive [10,14], then a negative correlation [5,8] between COVID-19 infection and population density, this study was an opportunity to further investigate this issue, by using very detailed data, and a more rigorous analytical approach. Our results confirm evidence of contrasting statistical correlations and highlight the impact of the scale of measurement on these correlations. On the one hand, COVID-19 infection was found to be significantly negatively associated with population density when no further adjustments were made and when population density was calculated using the total area of the spatial units as the denominator. On the other hand, when using adjusted population density, which considers only the built-up area as the denominator, a significant positive association with COVID-19 infection was observed. Therefore, adjusting population density before its application in our spatial statistical analysis has addressed issues related to the intrinsic properties of raw, non-adjusted population density such as the implicit assumption of uniformity over spatial units [13], the varying definitions of boundaries and the effect of varying scale of measurements [61]. Since the early stages of the pandemic, areas with high population density have experienced the greatest burden of COVID-19 infections [62,63]. Population density has significantly influenced the spatial distribution of COVID-19 [64], and has been a key indicator for implementing

control measures such as physical distancing [65] that aimed to combat the spread of the disease. Our findings link negative correlation between COVID-19 infection and population density to the omission of important methodological considerations. Conversely, the positive association that emerges following further adjustements confirms the role of population density as a primary risk factor for infectious diseases [61], including COVID-19 diseases.

Similarly, we found a significant positive association between residential areas and COVID-19 infection. In contrast, COVID-19 infection was negatively associated with non-residential areas, which include ACS areas. The distance to marketplaces was found to be not significant, suggesting that the effect of mobility was not fully captured by the geospatial predictors used in this study. Despite the complexities surrounding the influence of neighborhood features on the spread of COVID-19 [66], the positive association between COVID-19 infection and residential areas provides insights into local exposure in relation to built-up areas and land use characteristics. Additional information about residential dwellers, such as household social composition and income levels, could further reveal significant disparities in the spread of COVID-19 across residential areas [67].

The spatiotemporal model with space-time interaction demonstrated higher predictive performance compared to the non-spatial model and the spatiotemporal model with separable space-time effects. Including the space-time interaction improved the model performance as it accounts for specific local spikes, which cannot be explained by overall spatial or temporal effects alone. Indeed, the spatiotemporal interaction term has the benefit of capturing localised deviations in the infection risk, which is particularly valuable for identifying transient, area-specific surges, thereby enhancing model's sensitivity to local heterogeneity [40,41].

There is an important limitation that deserves to be highlighted. The epidemiological data provided by the Ministry of Health includes only the location of cases resulting from community transmission, therefore total case counts in some areas could be underrepresented. No information is available on the spatial distribution of other types of infection, such as the so-called "contact cases". Including these in the analysis could have refined our results. Furthermore, despite the low mortality rate from COVID-19, data on COVID-19 related-deaths could have provided additionnal insigths into the role of contextual risk factors in the spread of the disease. In this study, we aggregated the number of confirmed infection by months, which in turn smooths the daily reporting noise. Nonetheless, missing data, under-reporting and uneven testing across neighbourhoods may still affect the results. For instance, areas with better testing access might report more cases, which could confound the association with contextual risk factors. Potential data biases may arise from incomplete reporting or spatial variations in the detection of the infection, although greater concentration of health facilities and shared testing infrastructure in Dakar may limit these differences. Lastly, it is worth mentioning that, although remotely-sensed variables are useful proxies that capture meaningful landscape characteristics, census or survey-based household-level crowding and socioeconomic variables are useful indicators that could further refine our spatiotemporal model, and improve our understanding on the influence of contextual risk factors on COVID-19 infection.

5. Conclusion

The COVID-19 related social, health and economic impact has been gradually lessened, thanks to various efforts notably the rapid deployment of various vaccines that prompted a high level of population immunity. But in the post pandemic era, the disease is still circulating and the end of the

global health emergency has led to the emergence of a global health threat. The risk of COVID-19 infection, or re-infection persists, as new virus variants can emerge, causing new surges in cases and deaths.

From a public health perspective, the research findings gained from this study can be valuable resources for health systems in low- and middle-income countries, for geographically targeted interventions. These outputs can serve as support tools for the optimised distribution and less-labour intensive delivery of the COVID-19 vaccine. They can support disease surveillance system through~~and~~ the monitoring of the potential COVID-19 outbreaks. Testing or vaccination efforts should prioritize high-risk western neighbourhoods, or dense residential areas.

Furthermore, the model can be adapted for the study of other infectious diseases, including malaria, tuberculosis and other neglected tropical diseases. Here we can leverage the flexibility of Bayesian spatiotemporal models, which has gained more popularity due to their capacity to incorporate spatial and temporal dependencies, uncertainties and intricate interactions. Bayesian computing has long been a complex task, but in the era of big data and the rapid advancement of computer technology, the Bayesian framework has been enhanced enough to allow in-depth investigations of epidemiological data, and the study of different diseases with a different and more extensive set of regressors.

Acknowledgment

This work was supported, in whole or in part, by the Gates Foundation under the GRID3 project. The conclusions and opinions expressed in this work are those of the author(s) alone and shall not be attributed to the Foundation. Under the grant conditions of the Foundation, a Creative Commons Attribution 4.0 License has already been assigned to the Author Accepted Manuscript version that might arise from this submission. Please note works submitted as a preprint have not undergone a peer review process.

The authors gratefully thanks the ASSESS project, funded by the ARES-CDD (https://www.ares-ac.be/fr), which provided the access to the neighbourhood's shapefile used as base layer to produce Fig 1, and 2 as well as S1 Fig and S6 Fig.

Data availability

The epidemiological data used in this study is publicly available through a request from the Ministry of Health. The geospatial predictors that we utilised to perform the analyses are freely available through the WorldpPop (https://www.worldpop.org/datacatalog/) and MAUPP data repositories (https://maupp.ulb.ac.be/page/grippa2018/). The neighbourhood shapefile used as base layer is accessible upon request through the following repository (distributed under CC-BY license): https://github.com/assanenian/INLA_Bayesian_Spatiotemporal

Code availability

All the code used to support this research, as well as the R-shiny app we developed to visualise the results are available in the following repository:

https://github.com/assanenian/INLA_Bayesian_Spatiotemporal

Supporting information

S1 Fig. SPDE triangulation of the study area

S2 Fig. Marginal posterior distributions of covariate coefficients for the separable spatio-temporal model

S3 Fig. Marginal posterior distributions of covariate coefficients for the spatiotemporal interaction model

S4 Fig. Marginal posterior distribution of the random effects components for the separable spatiotemporal model. Top: Marginal posterior mean of the spatial random effect $\mathbf{S}(.)$
Middle: Marginal posterior mean of the iid effect $\mathbf{u_i}$
Bottom: Marginal posterior mean of the temporal random effect $\boldsymbol{\gamma_t}$

S5 Fig. ***Marginal posterior distribution of the random effects components for the spatiotemporal interaction model. Top panel: Marginal posterior mean of the spatial random effect $S(.)$***
Second panel: Marginal posterior mean of the iid effect u_i
Third panel: Marginal posterior mean of the temporal random effect γ_t
Bottom panel: Spatiotemporal interaction effect

S6 Fig. Overview of the R-Shiny App that visualizes the comparison between the observed and predicted COVID-19 prevalence.

References

1. Coronavirus [Internet]. [cited 2025 Apr 2]. Available from: https://www.who.int/health-topics/coronavirus

2. Mathieu E, Ritchie H, Rodés-Guirao L, Appel C, Gavrilov D, Giattino C, et al. Coronavirus (COVID-19) Vaccinations. Our World in Data [Internet]. 2024 Nov 15 [cited 2024 Nov 22]; Available from: https://ourworldindata.org/covid-vaccinations

3. Ndwandwe D, Wiysonge CS. COVID-19 vaccines. Current Opinion in Immunology. 2021 Aug 1;71:111–6.

4. Cohen C, Pulliam J. COVID-19 infection, reinfection, and the transition to endemicity. The Lancet. 2023 Mar 11;401(10379):798–800.

5. Carozzi F, Provenzano S, Roth S. Urban Density and Covid-19 [Internet]. Rochester, NY: Social Science Research Network; 2020 [cited 2025 May 7]. Available from: https://papers.ssrn.com/abstract=3643204

6. Cleary E, Atuhaire F, Sorichetta A, Ruktanonchai N, Ruktanonchai C, Cunningham A, et al. Comparing lagged impacts of mobility changes and environmental factors on COVID-19 waves in rural and urban India: A Bayesian spatiotemporal modelling study. PLOS Global Public Health. 2025 Apr 30;5(4):e0003431.

7. Cordes J, Castro MC. Spatial analysis of COVID-19 clusters and contextual factors in New York City. Spatial and Spatio-temporal Epidemiology. 2020 Aug 1;34:100355.

8. Hamidi S, Sabouri ,Sadegh, and Ewing R. Does Density Aggravate the COVID-19 Pandemic?: Early Findings and Lessons for Planners. Journal of the American Planning Association. 2020 Oct 1;86(4):495–509.

9. Huang H, Shi H, Zordan M, Lo SM, Tsou JY. Investigating the Spatiotemporal Relationship between the Built Environment and COVID-19 Transmission. ISPRS International Journal of Geo-Information. 2023;12(10).

10. Kadi N, Khelfaoui M. Population density, a factor in the spread of COVID-19 in Algeria: statistic study. Bull Natl Res Cent. 2020;44(1):138.

11. Rojas-Quiroz J, Marmolejo-Duarte C. Determining Equality of Infection Rates: A Spatial Analysis of Factors Associated with the Spread of COVID-19 in Barcelona, Spain. Journal of Urban Planning and Development. 2022;148(3).

12. Boyce MR, Katz R, Standley CJ. Risk Factors for Infectious Diseases in Urban Environments of Sub-Saharan Africa: A Systematic Review and Critical Appraisal of Evidence. Tropical Medicine and Infectious Disease. 2019 Dec;4(4):123.

13. Bhadra A, Mukherjee A, Sarkar K. Impact of population density on Covid-19 infected and mortality rate in India. Model Earth Syst Environ. 2021 Mar 1;7(1):623–9.

14. Garland P, Babbitt D, Bondarenko M, Sorichetta A, Tatem AJ, Johnson O. The COVID-19 pandemic as experienced by the individual [Internet]. arXiv; 2020 [cited 2025 May 7]. Available from: http://arxiv.org/abs/2005.01167

15. Oztig LI, Askin OE. Human mobility and coronavirus disease 2019 (COVID-19): a negative binomial regression analysis. Public Health. 2020 Aug;185:364–7.

16. Lai S, Ruktanonchai NW, Zhou L, Prosper O, Luo W, Floyd JR, et al. Effect of non-pharmaceutical interventions to contain COVID-19 in China. Nature. 2020 Sep;585(7825):410–3.

17. Lai S, Ruktanonchai NW, Carioli A, Ruktanonchai CW, Floyd JR, Prosper O, et al. Assessing the Effect of Global Travel and Contact Restrictions on Mitigating the COVID-19 Pandemic. Engineering. 2021 Jul 1;7(7):914–23.

18. Lai S, Bogoch II, Ruktanonchai NW, Watts A, Lu X, Yang W, et al. Assessing spread risk of COVID-19 in early 2020. Data Science and Management. 2022 Dec 1;5(4):212–8.

19. López-Arellano O, Bañuelos-Moreno M, Moreno-Altamirano A, López-Moreno S, Garrido-Latorre F. Socio-territorial inequalities and Covid-19 mortality in Mexico City. Salud Publica de Mexico. 2024;66(5):699–707.

20. Sun F, Matthews SA, Yang TC, Hu MH. A spatial analysis of the COVID-19 period prevalence in U.S. counties through June 28, 2020: where geography matters? Ann Epidemiol. 2020 Dec;52:54-59.e1.

21. Vandentorren S, Smaïli S, Chatignoux E, Maurel M, Alleaume C, Neufcourt L, et al. The effect of social deprivation on the dynamic of SARS-CoV-2 infection in France: a population-based analysis. The Lancet Public Health. 2022;7(3):e240–9.

22. Nazia N, Butt ZA, Bedard ML, Tang WC, Sehar H, Law J. Methods Used in the Spatial and Spatiotemporal Analysis of COVID-19 Epidemiology: A Systematic Review. International Journal of Environmental Research and Public Health. 2022 Jan;19(14):8267.

23. Chen D. Modeling the Spread of Infectious Diseases: A Review. In: Analyzing and Modeling Spatial and Temporal Dynamics of Infectious Diseases [Internet]. John Wiley & Sons, Ltd; 2014 [cited 2024 Dec 1]. p. 19–42. Available from: https://onlinelibrary.wiley.com/doi/abs/10.1002/9781118630013.ch2

24. Niraula P, Mateu J, Chaudhuri S. A Bayesian machine learning approach for spatio-temporal prediction of COVID-19 cases. Stoch Environ Res Risk Assess. 2022 Aug 1;36(8):2265–83.

25. Actualites | MINISTÈRE DE LA SANTÉ ET DE L'ACTION SOCIALE [Internet]. [cited 2025 Apr 7]. Available from: https://www.sante.gouv.sn/actualites

26. Petit V, Robin N, Martin N. Spatialité et temporalité de l'épidémie de la Covid-19 au Sénégal. Le processus de production des données sanitaires au regard des discontinuités territoriales. Revue francophone sur la santé et les territoires [Internet]. 2021 Jun 17 [cited 2024 Oct 24]; Available from: https://journals.openedition.org/rfst/1150

27. Tizzoni M, Bajardi P, Decuyper A, King GKK, Schneider CM, Blondel V, et al. On the Use of Human Mobility Proxies for Modeling Epidemics. PLOS Computational Biology. 2014 Jul 10;10(7):e1003716.

28. Gadiaga AN, De Longueville F, Georganos S, Grippa T, Dujardin S, Diène AN, et al. Neighbourhood-level housing quality indices for health assessment in Dakar, Senegal. Geospat Health [Internet]. 2021 May 5 [cited 2023 Jul 7];16(1). Available from: https://www.geospatialhealth.net/index.php/gh/article/view/910

29. Borderon M, Oliveau S, Machault V, Vignolles C, Lacaux JP, N'Donky A. Qualifier les espaces urbains à Dakar, Sénégal. Cybergeo: European Journal of Geography [Internet]. 2014 Mar 27 [cited 2025 Mar 18]; Available from: https://journals.openedition.org/cybergeo/26250

30. Agence Nationale de la Statistique et de la Démographie (ANSD) du Sénégal | «Un centre d'excellence dans un Système statistique national fort» [Internet]. [cited 2025 Apr 9]. Available from: https://www.ansd.sn/

31. Tais Grippa, Stefanos Georganos. Dakar very-high resolution land cover map [Internet]. Zenodo; 2018 [cited 2018 Oct 9]. Available from: https://zenodo.org/record/1290800#.W7xWQ_a2w2w

32. Grippa T, Georganos S. Dakar Land Use Map At Street Block Level [Internet]. Zenodo; 2018 [cited 2018 Oct 8]. Available from: https://zenodo.org/record/1291389

33. Georganos S, Gadiaga AN, Linard C, Grippa T, Vanhuysse S, Mboga N, et al. Modelling the Wealth Index of Demographic and Health Surveys within Cities Using Very High-Resolution Remotely Sensed Information. Remote Sensing. 2019 Jan;11(21):2543.

34. Grippa T, Linard C, Lennert M, Georganos S, Mboga N, Vanhuysse S, et al. Improving Urban Population Distribution Models with Very-High Resolution Satellite Information. Data. 2019 Mar;4(1):13.

35. Lloyd CT, Chamberlain H, Kerr D, Yetman G, Pistolesi L, Stevens FR, et al. Global spatio-temporally harmonised datasets for producing high-resolution gridded population distribution datasets. Big Earth Data. 2019;3(2):108–39.

36. Marconcini M, Metz- Marconcini A, Esch T, Gorelick N. Understanding Current Trends in Global Urbanisation - The World Settlement Footprint Suite. giforum. 2021;1:33–8.

37. Akinwande MO, Dikko HG, Samson A. Variance Inflation Factor: As a Condition for the Inclusion of Suppressor Variable(s) in Regression Analysis. Open Journal of Statistics. 2015;05(07):754–67.

38. O'brien RM. A Caution Regarding Rules of Thumb for Variance Inflation Factors. Qual Quant. 2007 Oct 1;41(5):673–90.

39. Barnett AG, Dobson AJ. Analysing Seasonal Health Data [Internet]. Berlin, Heidelberg: Springer; 2010 [cited 2025 Oct 28]. (Statistics for Biology and Health). Available from: http://link.springer.com/10.1007/978-3-642-10748-1

40. Simpson D, Rue H, Riebler A, Martins TG, Sørbye SH. Penalising Model Component Complexity: A Principled, Practical Approach to Constructing Priors. Statistical Science. 2017 Feb;32(1):1–28.

41. Blangiardo M, Cameletti M, Baio G, Rue H. Spatial and spatio-temporal models with R-INLA. Spatial and Spatio-temporal Epidemiology. 2013 Dec 1;7:39–55.

42. Moraga P, Dean C, Inoue J, Morawiecki P, Noureen SR, Wang F. Bayesian spatial modelling of geostatistical data using INLA and SPDE methods: A case study predicting malaria risk in Mozambique. Spatial and Spatio-temporal Epidemiology. 2021 Nov 1;39:100440.

43. Rue H, Riebler A, Sørbye SH, Illian JB, Simpson DP, Lindgren FK. Bayesian Computing with INLA: A Review. Annual Review of Statistics and Its Application. 2017 Mar 7;4(Volume 4, 2017):395–421.

44. Lambert D. Zero-Inflated Poisson Regression, with an Application to Defects in Manufacturing. Technometrics. 1992;34(1):1–14.

45. Rose CE, Martin ,S. W., Wannemuehler ,K. A., and Plikaytis BD. On the Use of Zero-Inflated and Hurdle Models for Modeling Vaccine Adverse Event Count Data. Journal of Biopharmaceutical Statistics. 2006 Aug 1;16(4):463–81.

46. Chaudhuri S, Juan ,Pablo, and Mateu J. Spatio-temporal modeling of traffic accidents incidence on urban road networks based on an explicit network triangulation. Journal of Applied Statistics. 2023 Dec 10;50(16):3229–50.

47. Gilks WR. Markov Chain Monte Carlo. In: Encyclopedia of Biostatistics [Internet]. John Wiley & Sons, Ltd; 2005 [cited 2025 May 9]. Available from: https://onlinelibrary.wiley.com/doi/abs/10.1002/0470011815.b2a14021

48. Brooks S, Gelman A, Jones G, Meng XL, editors. Handbook of Markov Chain Monte Carlo. New York: Chapman and Hall/CRC; 2011. 619 p.

49. Rue H, Martino S, Chopin N. Approximate Bayesian Inference for Latent Gaussian models by using Integrated Nested Laplace Approximations. Journal of the Royal Statistical Society Series B: Statistical Methodology. 2009 Apr 1;71(2):319–92.

50. Lindgren F, Rue H, Lindström J. An Explicit Link between Gaussian Fields and Gaussian Markov Random Fields: The Stochastic Partial Differential Equation Approach. Journal of the Royal Statistical Society Series B: Statistical Methodology. 2011 Sep 1;73(4):423–98.

51. Krainski E, Gómez-Rubio V, Bakka H, Lenzi A, Castro-Camilo D, Simpson D, et al. Advanced Spatial Modeling with Stochastic Partial Differential Equations Using R and INLA. New York: Chapman and Hall/CRC; 2018. 298 p.

52. Matérn B. Spatial variation. 2nd ed. Berlin, New York: Springer-Verlag; 1986. 151 p.

53. Juan Verdoy P. Enhancing the SPDE modeling of spatial point processes with INLA, applied to wildfires. Choosing the best mesh for each database. Communications in Statistics - Simulation and Computation. 2021 Oct 3;50(10):2990–3030.

54. Beginner’s Guide to Spatial, Temporal and Spatial-Temporal Ecological Data Analysis with R-INLA. Volume 1 [Internet]. [cited 2025 May 9]. Available from: https://www.highstat.com/index.php/books2?view=article&id=11&catid=18

55. Aswi A, Cramb SM, Moraga P, Mengersen K. Bayesian spatial and spatio-temporal approaches to modelling dengue fever: a systematic review. Epidemiol Infect. 2018 Oct 29;147:e33.

56. Cheng J, Bambrick H, Tong S, Su H, Xu Z, Hu W. Winter temperature and myocardial infarction in Brisbane, Australia: Spatial and temporal analyses. Science of the Total Environment [Internet]. 2020 May 1 [cited 2024 Nov 19];715(136860). Available from: http://www.scopus.com/inward/record.url?scp=85078777422&partnerID=8YFLogxK

57. Georganos S, Brousse O, Dujardin S, Linard C, Casey D, Milliones M, et al. Modelling and mapping the intra-urban spatial distribution of Plasmodium falciparum parasite rate using very-high-resolution satellite derived indicators. Int J Health Geogr. 2020 Sep 21;19(1):38.

58. Guégan JF, Vargas Campos CA, Chevillon C, Sylla A, Dogbe M, Fast KM, et al. Land-use and land-cover changes and emerging non-tuberculous and tuberculous infectious diseases in humans and animals: a mixed-methods review of research findings from global and spatio-temporal perspectives. Environ Res: Health. 2025 Feb;3(1):012003.

59. Myer MH, Johnston JM. Spatiotemporal Bayesian modeling of West Nile virus: Identifying risk of infection in mosquitoes with local-scale predictors. Science of The Total Environment. 2019 Feb 10;650:2818–29.

60. Samrat A, Purse BV, Vanak A, Chaudhary A, Uday G, Rahman M, et al. Producing context specific land cover and land use maps of human-modified tropical forest landscapes for infectious disease applications. Science of The Total Environment. 2024 Feb 20;912:168772.

61. Paez A, Lopez FA, Menezes T, Cavalcanti R, Pitta MG da R. A Spatio-Temporal Analysis of the Environmental Correlates of COVID-19 Incidence in Spain. Geographical Analysis. 2021;53(3):397–421.

62. Salem G. La santé dans la ville: géographie d'un petit espace dense : Pikine (Sénégal). KARTHALA Editions; 1998. 420 p.

63. Ribeiro HV, Sunahara AS, Sutton J, Perc M, Hanley QS. City size and the spreading of COVID-19 in Brazil. PLoS One. 2020;15(9):e0239699.

64. Stier AJ, Berman MG, Bettencourt LMA. Early pandemic COVID-19 case growth rates increase with city size. npj Urban Sustain. 2021 Jun 28;1(1):1–6.

65. Wong DWS, Li Y. Spreading of COVID-19: Density matters. PLOS ONE. 2020 Dec 23;15(12):e0242398.

66. Chamberlain HR, Lazar AN, Tatem AJ. High-resolution estimates of social distancing feasibility, mapped for urban areas in sub-Saharan Africa. Sci Data. 2022 Nov 18;9(1):711.

67. Zhang X, Sun Z, Ashcroft T, Dozier M, Ostrishko K, Krishan P, et al. Compact cities and the Covid-19 pandemic: Systematic review of the associations between transmission of Covid-19 or other respiratory viruses and population density or other features of neighbourhood design. Health & Place. 2022 Jul 1;76:102827.

68. Hong B, Bonczak BJ, Gupta A, Thorpe LE, Kontokosta CE. Exposure density and neighborhood disparities in COVID-19 infection risk. Proceedings of the National Academy of Sciences. 2021 Mar 30;118(13):e2021258118.

Author contributions

Conceptualisation, methodology: ANG, MWT, AND; formal analysis and validation: AND and SC; data curation: MWT; Writing and editing: ANG, SC, NS, CL, SL, EC, CCN, AL, AJT, OY; supervision: AND.

Competing interests

No competing interests to declare

Response to Reviewer's comments

1. The manuscript would benefit from a clearer and more detailed description of the modeling methods/tools, assumptions, and numerical implementation.
The rationale for using a temporal Poisson regression framework in Model 1, and spatiotemporal approaches in Models 2 and 3 should be explained. The authors should clarify the motivation for constructing multiple models and whether direct comparisons between them are appropriate. Additionally, details on how the simulations were numerically implemented are needed to ensure reproducibility.

Response: Thank you for your comment. To ensure reproducibility of the results, the code used to support this research, as well as the R-shiny app we developed to visualise the results are publicly available in a dedicated GitHub repository. We have added this additional text in the manuscript to explain the rationale for using temporal Poisson regression framework in Model 1, and spatiotemporal approaches in Models 2 and 3.

New addition to the manuscript:

"The rationale for constructing a temporal regression and spatiotemporal approaches is that temporal patterns often occur in different aspects of health, particularly in infectious disease transmission. Furthermore, infectious disease diffusion and spread is an intrinsically spatial process, as it involves complex interactions between human and different landscape attributes. Therefore, the motivation for constructing multiple models in this study, and comparing them is that, in addition to temporal correlation, possible spatiotemporal correlation may play a crucial role, with the spatial correlation constant or changing over time." (lines 237 – 243)

2. The models rely on several assumptions, including assumed parameter values, priors, etc. How sensitive are the results to these assumptions? It would also be helpful to clearly outline the three models in the methodology, explicitly stating the assumptions for each, and distinguishing which parameters are estimated versus assumed. A summary table listing all parameters and their values would improve clarity.

Response: We thank the reviewer for this valuable comment. We acknowledge the importance of model assumptions and prior choices in Bayesian inference. In the current study, we have chosen to provide default prior distributions for all parameters in R-INLA, as these have been chosen partly based on priors commonly used in the literature (Martins et al. 2013; Blangiardo and Cameletti 2015; Rue et al. 2016; Moraga 2020). To ensure robustness, we tested several alternative but reasonable prior specifications and obtained consistent results across models, suggesting that our conclusions are not sensitive to small variations in prior settings.

In response to the other suggestion, we have added a detailed description of the three models and a summary table listing all key parameters, their definitions, priors and whether they were estimated or assumed. These additions appear in the Methods section (Section 3.2).

New addition to the manuscript:

"All models assume that the number of COVID-19 cases follows a Poisson likelihood, with the expected count determined by population size and relative risk. The models differ in how spatial and temporal dependencies are incorporated. The temporal model includes monthly random effects modelled as either RW1 or RW2 processes, while the spatiotemporal models incorporate both spatially structured effects (using SPDE approach) and temporally structured effects (RW1). The interaction model further includes an independent spatiotemporal interaction term to capture unobserved localized deviations. Estimated parameters are inferred from the data through posterior distributions and include the regression coefficients (β), IID random effects (u_i), temporal effects (γ_t), spatiotemporal interactions (δ_{it}), and spatial field values ($S(x_i)$). Assumed parameters are chosen based on prior knowledge or literature, such as the parameters of the PC priors ([0.5, 0.01] for u_i, [1, 0.01] for γ_t) or the prior for the SPDE spatial range (Simpson et al., 2017). Fixed parameters are held constant and define model structure, such as the Matérn smoothness parameter v and the spatial dimension d (Lindgren et al., 2011). A summary of estimated parameters and priors is presented in table 2." (lines 372 – 386)

Parameter	Description	Model(s) Used	Prior Distribution
β_o, β	Fixed effects (intercept, covariates)	All models	Normal (0, 0.001)
u_i	IID random effect (neighbourhood-level variability)	Spatiotemporal	PC prior [0.5, 0.01]
γ_t	Temporal random effect (RW1 or RW2)	Temporal, Spatiotemporal	PC prior [1, 0.01]
δ_{it}	Spatiotemporal interaction term	Interaction model only	Normal (0, 1/τδ)
$S(x_i)$	Spatially structured effect (SPDE)	Spatiotemporal	Matérn covariance, PC prior [1, 0.1]
τ	Precision parameters (for random effects)	All models	Inverse Gamma (1, 0.00005)

ν	Smoothness parameter in SPDE	Spatiotemporal	Fixed at 1
κ	Scale parameter in SPDE	Spatiotemporal	Derived from range r

3. In Section 2.2.2, the construction of the covariate matrices and the spatially structured random effects S(·) is unclear. Provide details on how these were formulated. It may also be good to mention the number of neighborhoods included in the analysis.

Response: The spatially structured random effects $S(\cdot)$ were constructed using the stochastic partial differential equation (SPDE) approach, which provides the representation of a Gaussian random field with Matérn covariance (Lindgren, Rue & Lindström, 2011). In this framework, the continuous field is defined as the solution to

$$(\boldsymbol{\kappa}^2 - \boldsymbol{\Delta})^{(\alpha/2)}(\boldsymbol{\tau S}) = \boldsymbol{W} \text{ on } \mathbb{R}^d$$

In practice, we used the inla.mesh.2d function from R-INLA package to implement this. Therefore, we added this additional text in the manuscript to explain how the covariate matrices and the spatially structured random effect $S(.)$ have been formulated.

New additions to the manuscript:

"The matrix of covariates was built by stacking the covariate values for every space-time combination, and separate random effect mapping matrices link these observations to spatial, temporal or spatiotemporal latent effects." (lines 302 – 304)

"the triangulated mesh has been generated using inla.mesh.2d function from R-INLA package, based on Delaunay triangulation method, with the best fitting mesh having 7365 vertices for the 1078 neighbourhoods considered in this study." (lines 364-367)

4. Clarify which quantities were fitted during the model fitting process and describe how this was done. Further details are needed on the use of INLA and SPDE, how these were implemented to obtain the results.

Response: Thank you for your comment. We have added this additional text in the manuscript to give more details about the model fitting process.

New addition to the manuscript:

"Estimated parameters are inferred from the data through posterior distributions and include the regression coefficients (β), IID random effects (u_i), temporal effects (γ_t),

spatiotemporal interactions (δ_{it}), and spatial field values ($S(x_i)$). Assumed parameters are chosen based on prior knowledge or literature, such as the parameters of the PC priors ([0.5, 0.01] for u_i, [1, 0.01] for γ_t) or the prior for the SPDE spatial range. Fixed parameters are held constant and define model structure, such as the Matérn smoothness parameter ν and the spatial dimension." (lines 378 – 384)

5. The clarity of the results in Table 2 should be improved, as it currently does not provide meaningful interpretation. For instance, what does a (negative) -0.003 mean population density represent? What does intercept refer to in the model and what does it describe? What does 1.25 mean residential areas represent?

Response: Thank you for your comment. We have added the additional text below in the manuscript to improve the clarity of the results in table 3.

"As the proportion of non-residential areas, and the count of people per hectare per neighbourhood's total area increase, the expected mean of the COVID-19 infection decreases by 0.003 and 0.67, respectively. These expected values fall within the 95% credible interval for both the effect of non-residential areas (-1.29, -0.04) and population density (-0.003, -0.002). Adjusted population density and residential areas exhibit a significant positive effect, indicating a strong positive influence. When the proportion of residential areas, and the count of people per built-up area increase, the expected mean of COVID-19 infection increases by 0.29 and 1.25, respectively. Also, there is a 95% probability this effect lies between 0.66 and 1.83 for residential areas, and between 0.24 and 0.34 for adjusted population density. For both spatiotemporal models, the fixed effects demonstrate a similar trend and nature." (lines 429 – 437)

6. It is unclear whether the results in Table 2 are derived from the model fitting. The manuscript may benefit from a figure illustrating how well the best fit model compare with the observed data.

Response: Thank you for your comment. We assessed the goodness of fit of the model by comparing the predicted COVID-19 infection derived from the best-fitting model against the observed COVID-19 infection. Figure 4 illustrate this comparison. We improved the clarity of this sentence in the results section to better highlight this aspect.

New addition to the manuscript:

"Fig 4 reports the comparison between predicted COVID-19 infections derived from the best-fitting model and observed data. The Pearson correlation coefficient is 97%, which indicates a strong positive association between the predicted and the observed COVID-19 infections." (lines 481 – 482)

7. Non-pharmaceutical interventions (NPIs) such as social distancing or school closures substantially changed human behavior and hence, are expected to have a significant effect on the COVID-19 transmission during the study period. How are NPIs accounted for in the modeling framework?

Response: Thank you for your comment. Human behaviour can influence disease transmission, and NPIs measures such as social distancing played a crucial role during the pandemic, as a public health measure implemented to combat the spread of COVID-19 infection. Therefore, this should be accounted for when modelling contextual factors associated to COVID-19 disease. In this study, we have used population density, which acts as a proxy for different aspects related to NPIs. We also explored the commonly-used Oxford covid response tracker data, but they are not spatially granular enough for this study. We further tried to address this point by incorporating the 'ease of social distancing index' variable. The ease of social distancing index (Chamberlain et al. 2022) is an NPI metric, that has been developed to help identify locations where social distancing in urban areas is likely to be very difficult. It has been added as geospatial covariates in the modelling workflow, but collinearity checks revealed redundancy issue of the ease social distancing index with population density, adjusted population density and built-up density. In fact, ease social distancing index was constructed based on population density and built-up density already considered in our model. As a result, it was further dropped to avoid multicollinearity effect in capturing the relationship between COVID-19 infection and potential risk factors. Following such change made in the study design, we updated the list of the geospatial covariates considered in this study, the table listing and describing them as well as the results of the statistical analyses performed to check for multicollinearity.

**Variable**	**Description**	**Source**
**Temperature**	Annual average temperature	WorldPop
**Built-up density**	Built-up area per total area	MAUPP[a]
**Distance to marketplaces**	Average distance to marketplaces	WorldPop
**Population density**	Count of people per total area	MAUPP
**Adjusted population density**	Count of people per built-up area	MAUPP
**Residential areas**	Proportion of land use (LU) used for residential purposes	MAUPP

**Non-residential ACS areas**	Proportion of LU without residences used for administrative, commercial and services (ACS) purposes	MAUPP
**Ease of social distancing index**	Score index that evaluates the feasibility of social distancing measure	WorldPop

New addition to the manuscript:

"Through a spatio-temporal Bayesian framework we evaluate the impact of population density, temperature, distance to marketplaces, non-pharmaceutical interventions such as ease of social distancing index, and residential characteristics on COVID-19 spread." (lines 103 – 105)

"Additionally, distance to marketplaces, ease of social distancing index, and temperature data obtained from the WorldPop data repository were used." (line 170)

"Among the 8 variables considered for this analysis and introduced in Table 1, built-up density and ease of social distancing index were found showing multicollinearity issue and further dropped." (lines 202 – 204)

8. One motivation for the modeling study is to provide targeted interventions, once the drivers of the disease spread are identified. Based on the results, provide insights into what specific targeted interventions may or might have helped mitigate COVID-19 transmission in the study area.

Response: Thank you for your comment. Specific public health interventions that can be driven by outputs gained from this study are identifying high risk areas that should be monitored for future potential COVID-19 outbreaks. Other safety measures can be the availability of the COVID-19 vaccines in those areas. And the manuscript already identified high-risk western neighbourhoods, or dense residential areas, as should be identified as priority areas, as part of testing or vaccination efforts. We further improved the clarity of this paragraph in the conclusion section.

New addition to the manuscript:

"From a public health perspective, the research findings gained from this study can be valuable resources for health systems in low- and middle-income countries, for geographically targeted interventions. These outputs can serve as support tools for the optimised distribution and less-labour intensive delivery of the COVID-19 vaccine. They can support disease surveillance system through the monitoring of the

potential COVID-19 outbreaks. Testing or vaccination efforts should prioritize high-risk western neighbourhoods, or dense residential areas.” (lines 571 – 576)
