# Supplementary material for: Spatio-temporal modelling of COVID-19 infection and associated risk factors in Dakar, Senegal
Source: PLOS Glob Public Health. 2026 Jun 11;6(6):e0004945. doi: 10.1371/journal.pgph.0004945 (PMC13257971; doi:10.1371/journal.pgph.0004945)
Supplement: Attachment — Submitted filename: Response to Reviewers.docx [file pgph.0004945.s007.docx]

**Response to Reviewer’s comments**

1. The manuscript would benefit from a clearer and more detailed description of the modeling methods/tools, assumptions, and numerical implementation.
The rationale for using a temporal Poisson regression framework in Model 1, and spatiotemporal approaches in Models 2 and 3 should be explained. The authors should clarify the motivation for constructing multiple models and whether direct comparisons between them are appropriate. Additionally, details on how the simulations were numerically implemented are needed to ensure reproducibility.

**Response: Thank you for your comment. To ensure reproducibility of the results****, the code used to support this research, as well as the R-shiny app we developed to visualise the results are publicly available in a dedicated** [**GitHub repository**](https://som-shinyapps.shinyapps.io/COVID19_Dakar/)**. We have added this additional text in the manuscript to explain the rationale for using temporal Poisson regression framework in Model 1, and spatiotemporal approaches in Models 2 and 3.**

New addition to the manuscript:

*“The rationale for constructing a temporal regression and spatiotemporal approaches is that temporal patterns often occur in different aspects of health, particularly in infectious disease transmission. Furthermore, infectious disease diffusion and spread is an intrinsically spatial process, as it involves complex interactions between human and different landscape attributes. Therefore, the motivation for constructing multiple models in this study, and comparing them is that, in addition to temporal correlation, possible spatiotemporal correlation may play a crucial role, with the spatial correlation constant or changing over time.” (lines 237 – 243)*

2. The models rely on several assumptions, including assumed parameter values, priors, etc. How sensitive are the results to these assumptions? It would also be helpful to clearly outline the three models in the methodology, explicitly stating the assumptions for each, and distinguishing which parameters are estimated versus assumed. A summary table listing all parameters and their values would improve clarity.

**Response: We thank the reviewer for this valuable comment. We acknowledge the importance of model assumptions and prior choices in Bayesian inference. In the current study, we have chosen to provide default prior distributions for all parameters in R-INLA, as these have been chosen partly based on priors commonly used in the literature (Martins et al. 2013; Blangiardo and Cameletti 2015; Rue et al. 2016; Moraga 2020). To ensure robustness, we tested several alternative but reasonable prior specifications and obtained consistent results across models, suggesting that our conclusions are not sensitive to small variations in prior settings.**

**In response to the other suggestion, we have added a detailed description of the three models and a summary table listing all key parameters, their definitions, priors and whether they were estimated or assumed. These additions appear in the Methods section (Section 3.2).**

New addition to the manuscript:

*“All models assume that the number of COVID-19 cases follows a Poisson likelihood, with the expected count determined by population size and relative risk. The models differ in how spatial and temporal dependencies are incorporated. The temporal model includes monthly random effects modelled as either RW1 or RW2 processes, while the spatiotemporal models incorporate both spatially structured effects (using SPDE approach) and temporally structured effects (RW1). The interaction model further includes an independent spatiotemporal interaction term to capture unobserved localized deviations. Estimated parameters are inferred from the data through posterior distributions and include the regression coefficients (β), IID random effects (uᵢ), temporal effects (γₜ), spatiotemporal interactions (δᵢₜ), and spatial field values (S(xᵢ)). Assumed parameters are chosen based on prior knowledge or literature, such as the parameters of the PC priors ([0.5, 0.01] for uᵢ, [1, 0.01] for γₜ) or the prior for the SPDE spatial range (Simpson et al., 2017). Fixed parameters are held constant and define model structure, such as the Matérn smoothness parameter ν and the spatial dimension d (Lindgren et al., 2011). A summary of estimated parameters and priors is presented in table 2.” (lines 372 – 386)*

| **Parameter** | **Description** | **Model(s) Used** | **Prior Distribution** |
| --- | --- | --- | --- |
| β₀, β | Fixed effects (intercept, covariates) | All models | Normal (0, 0.001) |
| uᵢ | IID random effect (neighbourhood-level variability) | Spatiotemporal | PC prior [0.5, 0.01] |
| γₜ | Temporal random effect (RW1 or RW2) | Temporal, Spatiotemporal | PC prior [1, 0.01] |
| δᵢₜ | Spatiotemporal interaction term | Interaction model only | Normal (0, 1/τδ) |
| S(xᵢ) | Spatially structured effect (SPDE) | Spatiotemporal | Matérn covariance, PC prior [1, 0.1] |
| τ | Precision parameters (for random effects) | All models | Inverse Gamma (1, 0.00005) |
| ν | Smoothness parameter in SPDE | Spatiotemporal | Fixed at 1 |
| κ | Scale parameter in SPDE | Spatiotemporal | Derived from range r |

3. In Section 2.2.2, the construction of the covariate matrices and the spatially structured random effects S(⋅) is unclear. Provide details on how these were formulated. It may also be good to mention the number of neighborhoods included in the analysis.

**Response: The spatially structured random effects** $\boldsymbol{S(\cdot)}$ **were constructed using the stochastic partial differential equation (SPDE) approach, which provides the representation of a Gaussian random field with Matérn covariance (Lindgren, Rue & Lindström, 2011). In this framework, the continuous field is defined as the solution to**

$$\left( \boldsymbol{\kappa}^{\boldsymbol{2}}\boldsymbol{-}\boldsymbol{\Delta} \right)^{\left( \boldsymbol{\alpha}/\boldsymbol{2} \right)}\left( \boldsymbol{\tau S} \right)\boldsymbol{=W}\mathbf{on}\mathbb{R}^{\boldsymbol{d}}$$

**In practice, we used the inla.mesh.2d function from R-INLA package to implement this. Therefore, we added this additional text in the manuscript to explain how the covariate matrices and the spatially structured random effect** $\boldsymbol{S(.)}$ **have been formulated.**

New additions to the manuscript:

*“The matrix of covariates was built by stacking the covariate values for every space-time combination, and separate random effect mapping matrices link these observations to spatial, temporal or spatiotemporal latent effects.” (lines 302 – 304)*

*“the triangulated mesh has been generated using inla.mesh.2d function from R-INLA package, based on Delaunay triangulation method, with the best fitting mesh having 7365 vertices for the 1078 neighbourhoods considered in this study.” (lines 364-367)*

4. Clarify which quantities were fitted during the model fitting process and describe how this was done. Further details are needed on the use of INLA and SPDE, how these were implemented to obtain the results.

**Response: Thank you for your comment. We have added this additional text in the manuscript to give more details about the model fitting process.**

New addition to the manuscript:

*“Estimated parameters are inferred from the data through posterior distributions and include the regression coefficients (β), IID random effects (uᵢ), temporal effects (γₜ), spatiotemporal interactions (δᵢₜ), and spatial field values (S(xᵢ)). Assumed parameters are chosen based on prior knowledge or literature, such as the parameters of the PC priors ([0.5, 0.01] for uᵢ, [1, 0.01] for γₜ) or the prior for the SPDE spatial range. Fixed parameters are held constant and define model structure, such as the Matérn smoothness parameter ν and the spatial dimension.” (lines 378 – 384)*

5. The clarity of the results in Table 2 should be improved, as it currently does not provide meaningful interpretation. For instance, what does a (negative) -0.003 mean population density represent? What does intercept refer to in the model and what does it describe? What does 1.25 mean residential areas represent?

**Response: Thank you for your comment. We have added the additional text below in the manuscript to improve the clarity of the results in table 3.**

*“As the proportion of non-residential areas, and the count of people per hectare per neighbourhood’s total area increase, the expected mean of the COVID-19 infection decreases by 0.003 and 0.67, respectively. These expected values fall within the 95% credible interval for both the effect of non-residential areas (-1.29, -0.04) and population density (-0.003, -0.002). Adjusted population density and residential areas exhibit a significant positive effect, indicating a strong positive influence. When the proportion of residential areas, and the count of people per built-up area increase, the expected mean of COVID-19 infection increases by 0.29 and 1.25, respectively. Also, there is a 95% probability this effect lies between 0.66 and 1.83 for residential areas, and between 0.24 and 0.34 for adjusted population density. For both spatiotemporal models, the fixed effects demonstrate a similar trend and nature.” (lines 429 – 437)*

6. It is unclear whether the results in Table 2 are derived from the model fitting. The manuscript may benefit from a figure illustrating how well the best fit model compare with the observed data.

**Response: Thank you for your comment. We assessed the goodness of fit of the model by comparing the predicted COVID-19 infection derived from the best-fitting model against the observed COVID-19 infection. Figure 4 illustrate this comparison. We improved the clarity of this sentence in the results section to better highlight this aspect.**

New addition to the manuscript:

*“Fig 4 reports the comparison between predicted COVID-19 infections derived from the best-fitting model and observed data. The Pearson correlation coefficient is 97%, which indicates a strong positive association between the predicted and the observed COVID-19 infections.” (lines 481 – 482)*

7. Non-pharmaceutical interventions (NPIs) such as social distancing or school closures substantially changed human behavior and hence, are expected to have a significant effect on the COVID-19 transmission during the study period. How are NPIs accounted for in the modeling framework?

**Response: Thank you for your comment. Human behaviour can influence disease transmission, and NPIs measures such as social distancing played a crucial role during the pandemic, as a public health measure implemented to combat the spread of COVID-19 infection. Therefore, this should be accounted for when modelling contextual factors associated to COVID-19 disease. In this study, we have used population density, which acts as a proxy for different aspects related to NPIs. We also explored the commonly-used** [**Oxford covid response tracker data**](https://www.bsg.ox.ac.uk/research/covid-19-government-response-tracker)**, but they are not spatially granular enough for this study. We further tried to address this point by incorporating the ‘ease of social distancing index’ variable. The ease of social distancing index (Chamberlain et al. 2022) is an NPI metric, that has been developed to help identify locations where social distancing in urban areas is likely to be very difficult. It has been added as geospatial covariates in the modelling workflow, but collinearity checks revealed redundancy issue of the ease social distancing index with population density, adjusted population density and built-up density. In fact, ease social distancing index was constructed based on population density and built-up density already considered in our model. As a result, it was further dropped to avoid multicollinearity effect in capturing the relationship between COVID-19 infection and potential risk factors. Following such change made in the study design, we updated the list of the geospatial covariates considered in this study, the table listing and describing them as well as the results of the statistical analyses performed to check for multicollinearity.**

| **Variable** | **Description** | **Source** |
| --- | --- | --- |
| **Temperature** | Annual average temperature | WorldPop |
| **Built-up density** | Built-up area per total area | MAUPP^a^ |
| **Distance to marketplaces** | Average distance to marketplaces | WorldPop |
| **Population density** | Count of people per total area | MAUPP |
| **Adjusted population density** | Count of people per built-up area | MAUPP |
| **Residential areas** | Proportion of land use (LU) used for residential purposes | MAUPP |
| **Non-residential ACS areas** | Proportion of LU without residences used for administrative, commercial and services (ACS) purposes | MAUPP |
| **Ease of social distancing index** | Score index that evaluates the feasibility of social distancing measure | WorldPop |

New addition to the manuscript:

***“****Through a spatio-temporal Bayesian framework we evaluate the impact of population density, temperature, distance to marketplaces, non-pharmaceutical interventions such as ease of social distancing index, and residential characteristics on COVID-19 spread.” (lines 103 – 105)*

***“****Additionally, distance to marketplaces, ease of social distancing index, and temperature data obtained from the WorldPop data repository were used.” (line 170)*

***“****Among the 8 variables considered for this analysis and introduced in Table 1, built-up density and ease of social distancing index were found showing multicollinearity issue and further dropped.” (lines 202 – 204)*

8. One motivation for the modeling study is to provide targeted interventions, once the drivers of the disease spread are identified. Based on the results, provide insights into what specific targeted interventions may or might have helped mitigate COVID-19 transmission in the study area.

**Response: Thank you for your comment. Specific public health interventions that can be driven by outputs gained from this study are identifying high risk areas that should be monitored for future potential COVID-19 outbreaks. Other safety measures can be the availability of the COVID-19 vaccines in those areas. And the manuscript already identified high-risk western neighbourhoods, or dense residential areas, as should be identified as priority areas, as part of testing or vaccination efforts. We further improved the clarity of this paragraph in the conclusion section.**

New addition to the manuscript:

*“From a public health perspective, the research findings gained from this study can be valuable resources for health systems in low- and middle-income countries, for geographically targeted interventions. These outputs can serve as support tools for the optimised distribution and less-labour intensive delivery of the COVID-19 vaccine. They can support disease surveillance system through the monitoring of the potential COVID-19 outbreaks. Testing or vaccination efforts should prioritize high-risk western neighbourhoods, or dense residential areas.” (lines 571 – 576)*
