# Supplementary material for: Spatio-temporal modelling of COVID-19 infection and associated risk factors in Dakar, Senegal
Source: PLOS Glob Public Health. 2026 Jun 11;6(6):e0004945. doi: 10.1371/journal.pgph.0004945 (PMC13257971; doi:10.1371/journal.pgph.0004945)
Supplement: Attachment — Submitted filename: Response_to_reviewer.docx [file pgph.0004945.s009.docx]

**Reviewer’s comments**

1. I consider language & wording to be overall very good. There are some places where singular/plural is grammatically wrong, and at least one sentence that seems to be incomplete.

**Response:**

**Thank you for proofreading the manuscript and for making suggestion to improve the writing. Your suggestions for changes have been accepted, and the document has undergone another thorough proofreading to ensure that there are no subject/verb and singular/plural inconsistencies.**

2. Regarding the choice of parameters for the Bayesian model, you mainly motivate it by literature. I think it would yet be better to provide further information on the meaning of the parameter choices as some of them appear arbitrary without further context. For example, in line 356, one parameter is set to sqrt(8v)/k. What is the rationale behind this and what is the potential impact of this parameter?

**Response:**

**We thank the reviewer for the comment.**

**A summary table listing all the parameters and their corresponding values was provided to help the reader gain better understanding of the model parameters.**

**Regarding the relationship** $\boldsymbol{r=}\frac{\sqrt{\boldsymbol{8}\boldsymbol{v}}}{\boldsymbol{k}}$ **, it follows directly from the Matérn** **covariance function and defines the practical spatial range, that is, the distance at which spatial correlation decreases to approximately 0.1. Thus, this choice provides an interpretable link between** $\boldsymbol{\kappa}$ **and the decay of spatial dependence. In our case, fixing** $\boldsymbol{v}$ **= 1 implies that** $\boldsymbol{\kappa}$ **directly controls how quickly spatial correlation decreases with distance, affecting the smoothness and spatial extent of the latent field.**

**The impact of this parameter is that it directly controls the strength and spatial extent of dependence in the latent field. Smaller values of** $\boldsymbol{\kappa}$ **(resulting in a larger r) produce smoother spatial fields with long-range correlation, while larger values of** $\boldsymbol{\kappa}$ **(resulting in a smaller r) lead to more localised spatial variation. Therefore, this parameter strongly influences how spatial structure is represented in the model but does not affect the mean structure.**

**The spatial range parameters were adopted from the empirical simulation study conducted by Lindgren et al., where different values of** $\boldsymbol{\kappa}$ **and** $\boldsymbol{v}$ **were tested in order to find the optimal value of r. We added a sentence in the manuscript to further clarify this aspect.**

New addition to the manuscript:

*“It is worth noting that the spatial range parameters were adopted from the empirical study conducted by Lindgren et al.****”*** *(lines 376 – 377)*

3. Regarding the implementation, you provide the Github link, which is great. I checked the code and was a bit surprised that it had relatively few lines. In any case, I recommend listing the used R packages in the manuscript as well.

**Response:**

**Thank you for flagging this.**

**Not sure what was the cause, but we did check the** [**Github**](https://github.com/assanenian/INLA_Bayesian_Spatiotemporal/blob/main/dakar_covid_analysis.R) **repository to ensure that the script is here and can be viewed, and downloaded. Regarding the R packages used to carry out this work, I added this sentence at the end of the method section:**

New addition to the manuscript:

*“Specifically, the INLA package (https://www.r-inla.org/) was used to implement Bayesian regression modelling strategies described above. Model ouputs were subsequently extracted, postprocessed and visualised using INLAutils, the companion package to INLA, which provides specialised tools for model diagnostics and result interpretation.” (lines 433 – 437)*

4. Pacing: In several parts of the manuscript, there are long paragraphs, some of them with multiple ideas/aspects. Please consider inserting more line breaks. Also, in general, following the ideas along the main dimensions (what/why/how) could be improved.

**Response:**

**Thank you for your comment.**

**We have added several line breaks to resolve the issue of long paragraphs and preserve the smooth flow of the ideas. These changes were made to better distinguish the main dimensions of the study thereby improving the overall clarity and logical flow of the manuscript.**

5. Presentation of modeling approaches: You consider a range of different model (specifications). On first reading, I found it challenging to clearly distinguish between the considered models. I was wondering whether it would be good to present the models incl. their specifications in a table?

**Response:**

**Thank you for raising this important point.**

**Yes, I fully agree that a summary table would be very helpful for the readers to better understand and follow the description of the modelling strategies presented in the Methods section. As suggested, we have therefore added the summary table below.**

New addition to the manuscript: *(lines 229, and 257 – 258)*

| **Model** | **Type** | **Spatial structure** | **Space-time interaction** | **Model equation** | **Description** |
| --- | --- | --- | --- | --- | --- |
| Model 1 | Temporal | None | None | $\log\left( q_{t} \right)= \beta_{0}+ Z_{t}^{T}\beta+ u_{i}+ \gamma_{t}$ | Temporal AR model without spatial structure |
| Model 2 | Spatio-temporal | SPDE | Separable | $\log\left( q_{it} \right)= \beta_{0}+ Z_{it}^{T}\beta_{it}+ S\left( x_{i} \right)+ u_{i}+ \gamma_{t}$ | Combine AR temporal structure with spatial SPDE model and separate space and time effects (no interaction) |
| Model 3 | Spatio-temporal | SPDE | Yes | $\log\left( q_{it} \right)= \beta_{0}+ Z_{it}^{T}\beta_{it}+ S\left( x_{i} \right)+ u_{i}+ \gamma_{t}+ \delta_{it}$ | Combines AR temporal structure with spatial SPDE model and space-time interaction |

6. The variable qt is introduced as 'relative risk'. I think this should be defined more clearly; do you mean the probability of a transmission/infection given there is a contact to an infected person?

**Response:**

**Thank you for your comment.**

**Yes the variable** **qt, also known as relative risk, refers to the probability of infection given there is a contact to an infected person. We have added a sentence to further clarify the concept of relative risk in the present study.**

New addition to the manuscript:

*“the relative risk* $q_{t}$ *expresses the probability of infection given there is a contact to an infected person” (lines 273 – 274)*

7. The analysis is based on empiric/observational data and therefore causal interpretation of the modeling results is limited. You do not explicitly claim in the article that the results are directly to be understood as causal. However, I think it would be better to address the issue within the manuscript (discussion) that the model is not a causal model.

**Response:**

**Thank you for your comment.**

**This limitation deserves to be highlighted, along with the use of satellite-derived proxies of human presence/characteristics as predictors of COVID-19 infection. Accordingly, we have added the following sentences to the Discussion section of the manuscript.**

New addition to the manuscript:

***“****Notwithstanding the higher predictability of the spatiotemporal model, the link between COVID-19 infection and identified contextual factors may not be direct, as we are dealing with empiric observational data and satellite-derived variables that carry a certain degree of uncertainty. Consequently, caution is needed when drawing conclusions from the results, as causal inference from the model findings is limited****”*** *(lines 604 – 608)*

8. Regarding INLA, can you discuss in the manuscript what the drawbacks and potential risks are for the considered model? How reliable is the approximation for spatial data? Is it possible to do a (partial) robustness check by using an actual MCMC posterior simulation (or another method of more accurately representing the posterior distribution)?

**Response:**

**We thank the reviewer for raising this important point regarding the use of INLA. INLA relies on deterministic Laplace approximations to obtain marginal posterior distributions and therefore does not provide exact joint posterior samples as obtained from MCMC.
In the present study, the primary objective was to compare multiple temporal and spatiotemporal model specifications, including different random-effect structures and interaction terms. Performing full MCMC posterior simulation for each competing model would be computationally prohibitive given the size of the dataset and the complexity of the spatial field. For this reason, INLA was chosen as a practical and well-established alternative that enables systematic model comparison and sensitivity analysis within a reasonable computational time.**

New addition to the manuscript:

*“*It is worthy to mention that, INLA provides fast and accurate approximations for posterior marginal distributions in latent Gaussian models, but it does not generate exact joint posterior samples as in MCMC [41-43, 48]. The performance may be limited in models with highly non-Gaussian posteriors or complex dependence structures; however, for spatial and spatiotemporal Poisson models, the INLA-SPDE framework can yield results comparable to MCMC with substantially lower computational cost [42, 43, 50]. In particular, the SPDE-INLA framework has been extensively validated for spatial data and is known to produce results that closely match MCMC-based inference while being computationally much more efficient [41-43, 49, 50]. In the present study, the primary objective was to compare multiple temporal and spatiotemporal model specifications, including different random-effect structures and interaction terms. As a result, INLA was chosen to enable comparison of multiple competing model structures using alternative priors and model specifications.*” (lines 586 – 597)*

9. In lines 340 until 360, a rather complex series of parameters is introduced. Please specify more clearly in the manuscript why these are chosen (e.g., what is the motivation behind the Matern covariance) and which parameters are set by you and which are estimated within the model.

**Response:**

**We thank the reviewer for this helpful comment.**

**The Matérn covariance function was chosen because it provides a flexible and widely used framework for modelling spatial dependence, allowing explicit control over variance, smoothness, and spatial range. It is particularly suitable for spatial epidemiological data, where correlations typically decay smoothly with distance, and it is the standard choice within the SPDE–INLA framework due to its well-defined link between continuous Gaussian fields and discrete GMRFs as referred in [42, 43, 49, 50].**

New addition to the manuscript:

*“The Matérn covariance function was adopted because it provides a flexible and interpretable description of spatial dependence and is standard within the SPDE-INLA framework. Fixing the spatial dimension as 2 and the smoothness parameter as 1 to define the structure of the spatial field ensures computational stability [49, 50].” (lines 379 – 382)*
